# Supplementary material for: Procedural elements of the complete examination and screening of the healthy term neonate: a protocol for a scoping review and evidence map
Source: BMJ Paediatr Open. 2024 Jun 10;8(1):e002286. doi: 10.1136/bmjpo-2023-002286 (PMC11168178; doi:10.1136/bmjpo-2023-002286)
Supplement: Supplementary data [file bmjpo-2023-002286supp002.pdf]

Supplemental File 2 ScEvMap_CESoN Procedure Protocol

Contents

Appendix I The Components of the Complete Examination and Screening of the Healthy Term Neonate (CESoN) 1
Appendix II Reporting Guidelines PRISMA ScR Checklist (17) 4
Appendix III: Search strategy for both PubMed and CINAHL 6
Appendix IV Data Extraction Template - Coding Framework Headings and Tables 8
CESoN Procedural Elements Coding 8
Study Characteristics Coding 9

Appendix I The Procedural Elements of the Complete Examination and Screening of the Healthy Term Neonate (CESoN)

CESoN	Public Health England (PHE) [1]	National Institute of Health and Care Excellence (NICE) [2]	American Academy of Pediatrics [3]
**Prepare to Examine Neonate**	Ask: mother's medical and recent obstetric history, including any medication	Review the history.<br><br>Meconium passed within 24 hours of birth	Clinical course reveals no abnormalities that require continued hospitalization.<br><br>Review baby vital signs for preceding 12 hours.<br><br>Risk factors for sepsis?<br><br>Assess quality of mother-infant attachment and details of infant behaviour
	Check risk factors for eye disorders, CHD, DDH, and undescended testes/testis	Ask parents if they have any concerns about their baby's general wellbeing… or development.	has completed at least 2 successful feedings
	baby's family history	Ask parents if they have any concerns about their baby's …feeding	infant has urinated regularly and passed at least 1 stool
**Head-to-Toe Physical Examination and Screening**	baby's immediate postnatal health	Appearance, including colour, breathing, behaviour, activity and posture<br><br>Cry: assess sound.	Physical examination reveals no abnormalities that require continued hospitalization.<br><br>Check for jaundice.
		Head (including fontanelles), face, nose, mouth (including palate), ears, neck and general symmetry of head and facial features	
	Eyes: Asses position, structure, and red reflex.	Eyes: opacities, red reflex and colour of sclera	
		Neck and clavicles, limbs, hands, feet and digits; assess proportions and symmetry	
	Heart: Observation of	heart: position, heart rate, rhythm	

	tone, colour, size and shape of chest, respiratory rate and effort.<br>Palpation of femoral and brachial pulse, capillary refill time, chest, liver.<br>Auscultation of pulmonic, aortic, tricuspid, mitral, and midscapulae/coarctation areas.	and sounds, murmurs and femoral pulse volume	
		Lungs: respiratory effort, rate and lung sounds	
		abdomen: assess shape and palpate to identify any organomegaly; check condition of umbilical cord	
	(In males) Testes: Observe the scrotum for symmetry, size and colour. Palpation of scrotal sac (+inguinal canal and perineum if testis not located in scrotal sac).	Genitalia and anus: completeness and patency and undescended testes in boys	No evidence of excessive bleeding at the circumcision site at least 2 hours.
		Spine: inspect and palpate bony structures and check integrity of the skin	
		Skin: colour and texture as well as any birthmarks or rashes	
		Central nervous system: tone, behaviour, movements and posture; check newborn reflexes only if concerned	
	Hips: Observation of leg length, symmetry. Manipulation of each hip with Barlow and Ortolani test manoeuvres.	Hips: symmetry of the limbs, Barlow and Ortolani's manoeuvres	
		Measure weight and head circumference of babies in the first week	weigh the infant
**Relay Examination Findings, Baby Health Advice, Document Procedure and Findings.**		Advice on identifying and managing jaundice	Appropriate jaundice management and/or follow-up plans have been instituted
		Carry out/check bloodspot and hearing screening performed	Newborn metabolic and hearing screenings have been completed per hospital

			protocol
			Newborn pulse oximetry screening has been completed per hospital protocol
		Information to parents on baby care topics including skin, cord, feeding, bonding, signs and symptoms of illness, prevention of SIDS, Vitamin D supplementation, and immunisations.	training and information to mother about: breastfeeding, baby urine and stool patterns, baby care including cord, use of thermometer, signs symptoms of unwell baby, Immunisation, infant safety including car seat usage, prevention of SIDS, and hand hygiene

Appendix II Reporting Guidelines PRISMA ScR Checklist (17)

SECTION	ITEM	PRISMA-ScR CHECKLIST ITEM	REPORTED ON PAGE #
**TITLE**			
Title	1	Identify the report as a scoping review.	1
**ABSTRACT**			
Structured summary	2	Provide a structured summary that includes (as applicable): background, objectives, eligibility criteria, sources of evidence, charting methods, results, and conclusions that relate to the review questions and objectives.	1
**INTRODUCTION**			
Rationale	3	Describe the rationale for the review in the context of what is already known. Explain why the review questions/objectives lend themselves to a scoping review approach.	2
Objectives	4	Provide an explicit statement of the questions and objectives being addressed with reference to their key elements (e.g., population or participants, concepts, and context) or other relevant key elements used to conceptualize the review questions and/or objectives.	2
**METHODS**			
Protocol and registration	5	Indicate whether a review protocol exists; state if and where it can be accessed (e.g., a Web address); and if available, provide registration information, including the registration number.	2
Eligibility criteria	6	Specify characteristics of the sources of evidence used as eligibility criteria (e.g., years considered, language, and publication status), and provide a rationale.	3
Information sources*	7	Describe all information sources in the search (e.g., databases with dates of coverage and contact with authors to identify additional sources), as well as the date the most recent search was executed.	3,4
Search	8	Present the full electronic search strategy for at least 1 database, including any limits used, such that it could be repeated.	4,5
Selection of sources of evidence†	9	State the process for selecting sources of evidence (i.e., screening and eligibility) included in the scoping review.	5
Data charting process‡	10	Describe the methods of charting data from the included sources of evidence (e.g., calibrated forms or forms that have been tested by the team before their use, and whether data charting was done independently or in duplicate) and any processes for obtaining and confirming data from investigators.	5
Data items	11	List and define all variables for which data were sought and any assumptions and simplifications made.	5,6
Critical appraisal of individual sources of evidence§	12	If done, provide a rationale for conducting a critical appraisal of included sources of evidence; describe the methods used and how this information was used in any data synthesis (if appropriate).	6
Synthesis of results	13	Describe the methods of handling and summarizing the data that were charted.	Click here to enter text.
**RESULTS**			
Selection of sources of evidence	14	Give numbers of sources of evidence screened, assessed for eligibility, and included in the review, with reasons for exclusions at each stage, ideally using a flow diagram.	Click here to enter text.

Greene LM, *et al. BMJ Paediatrics Open* 2024; 8:e002286. doi: 10.1136/bmjpo-2023-002286

SECTION	ITEM	PRISMA-ScR CHECKLIST ITEM	REPORTED ON PAGE #
Characteristics of sources of evidence	15	For each source of evidence, present characteristics for which data were charted and provide the citations.	Click here to enter text.
Critical appraisal within sources of evidence	16	If done, present data on critical appraisal of included sources of evidence (see item 12).	Click here to enter text.
Results of individual sources of evidence	17	For each included source of evidence, present the relevant data that were charted that relate to the review questions and objectives.	Click here to enter text.
Synthesis of results	18	Summarize and/or present the charting results as they relate to the review questions and objectives.	Click here to enter text.
**DISCUSSION**			
Summary of evidence	19	Summarize the main results (including an overview of concepts, themes, and types of evidence available), link to the review questions and objectives, and consider the relevance to key groups.	Click here to enter text.
Limitations	20	Discuss the limitations of the scoping review process.	Click here to enter text.
Conclusions	21	Provide a general interpretation of the results with respect to the review questions and objectives, as well as potential implications and/or next steps.	Click here to enter text.
**FUNDING**			
Funding	22	Describe sources of funding for the included sources of evidence, as well as sources of funding for the scoping review. Describe the role of the funders of the scoping review.	7

Appendix III: Search strategy for both PubMed and CINAHL

Limits: *Years: 2013-2023; English language; Humans*

PubMed 2023 search strings keywords (abstract or title) and MeSH SHs:

P: “baby” OR “babies” OR “newborn” OR “newborns” OR “new-born” OR “new-borns” OR “neonate” OR “neonates” OR “neonatal” OR “infant” OR “infants” OR (MM "Infant, Newborn+") OR "infant, newborn"[MeSH Terms] OR "baby"[Title/Abstract] OR "babies"[Title/Abstract] OR "newborn"[Title/Abstract] OR "newborns"[Title/Abstract] OR "new-born"[Title/Abstract] OR "new-borns"[Title/Abstract] OR "neonate"[Title/Abstract] OR "neonates"[Title/Abstract] OR "neonatal"[Title/Abstract] OR "infant"[Title/Abstract] OR "infants"[Title/Abstract]

Concept string 1: "newborn infant physical examination" OR "NIPE" OR “head-to-toe” OR “head to toe” OR “clinical check-up” OR “check-up” OR “newborn exam*” OR “physical examination” OR “discharge examination” OR “examination before discharge” OR “clinical examination” OR “physical assessment” OR “physical check-up” OR "clinical assessment” OR (MM "Physical Examination+") OR ("Physical Examination/instrumentation"[Majr] OR "Physical Examination/methods"[Majr] OR "Physical Examination/standards"[Majr]) OR "newborn infant physical examination"[Title/Abstract] OR "NIPE"[Title/Abstract] OR "head-to-toe"[Title/Abstract] OR "check-up"[Title/Abstract] OR "checkup"[Title/Abstract] OR "newborn examination"[Title/Abstract] OR "physical examination"[Title/Abstract] OR "discharge examination"[Title/Abstract] OR "detailed physical"[Title/Abstract] OR "clinical examination"[Title/Abstract] OR "physical assessment"[Title/Abstract] OR "physical check-up"[Title/Abstract] OR "clinical assessment"[Title/Abstract] OR "developmental dysplasia of the hip/diagnosis"[MeSH Major Topic] OR "cataract/congenital"[MeSH Major Topic] OR "heart defects, congenital/diagnosis"[MeSH Major Topic] OR "cryptorchidism/diagnosis"[MeSH Major Topic] OR "neurologic examination/methods"[MeSH Major Topic] OR "Body Height"[MeSH Major Topic] OR "megalencephaly/diagnosis"[MeSH Major Topic] OR "microcephaly/diagnosis"[MeSH Major Topic] OR "Jaundice, Neonatal/diagnosis"[Majr]

Concept string 2: “performance” OR “procedural” OR “psychomotor skills” OR “technical performance” OR “technical skill” OR “evidence-based” OR “gold-standard” OR “gold standard” OR guidance OR guideline* OR standard* OR checklist OR consensus OR recommendation* OR error* OR omission OR mistake* OR "Psychomotor Performance"[Mesh] OR “Clinical Competence"[Mesh] OR "Evidence-Based Practice"[Mesh] OR "Psychomotor Performance"[MeSH Terms] OR "Clinical Competence"[MeSH Terms] OR "Evidence-Based Practice"[MeSH Terms] OR "Medical Errors"[MeSH Terms] OR "Guideline Adherence"[MeSH Terms] OR "Professional Competence/standards"[Mesh] OR "Standard of Care"[MeSH Terms] OR "performance"[Title/Abstract] OR "procedural"[Title/Abstract] OR "psychomotor skill*"[Title/Abstract] OR "technical performance"[Title/Abstract] OR "technical skill*"[Title/Abstract] OR "technique*"[Title/Abstract] OR "step*"[Title/Abstract] OR "evidence-based"[Title/Abstract] OR "gold-standard*"[Title/Abstract] OR "gold standard*"[Title/Abstract] OR guidance[Title/Abstract] OR guideline*[Title/Abstract] OR standard*[Title/Abstract] OR checklist*[Title/Abstract] OR consensus[Title/Abstract] OR recommendation*[Title/Abstract] OR error*[Title/Abstract] OR omission*[Title/Abstract] OR mistake*[Title/Abstract]

Context-

Greene LM, *et al. BMJ Paediatrics Open* 2024; 8:e002286. doi: 10.1136/bmjpo-2023-002286

CINAHL 2023 search strings keywords (abstract or title) and MeSH SHs:

Person: TI “baby” OR “babies” OR “newborn” OR “newborns” OR “new-born” OR “new-borns” OR “neonate” OR “neonates” OR “neonatal” OR “infant” OR “infants” OR (MM "Infant, Newborn+") OR AB “baby” OR “babies” OR “newborn” OR “newborns” OR “new-born” OR “new-borns” OR “neonate” OR “neonates” OR “neonatal” OR “infant” OR “infants” OR (MM "Infant, Newborn+")

Concept string 1 : TI "newborn infant physical examination" OR "NIPE" OR “head-to-toe” OR “head to toe” OR “clinical check-up” OR “check-up” OR “newborn exam*” OR “physical examination” OR “discharge examination” OR “examination before discharge” OR “clinical examination” OR “physical assessment” OR “physical check-up” OR "clinical assessment” OR (MM "Neonatal Assessment+") OR (MM "Physical Examination+") OR (MH "Developmental Dysplasia of the Hip/DI") OR (MH "Cataract/DI") OR (MH "Heart Defects, Congenital+/DI") OR (MH "Cryptorchidism/DI") OR (MH "Neurologic Examination/MT") OR (MH "Anthropometry/MT") OR (MH "Head Circumference/MT") OR (MH "Body Height/MT") OR (MH "Microcephaly/DI") OR (MH "Jaundice, Neonatal") OR AB "newborn infant physical examination" OR "NIPE" OR “head-to-toe” OR “head to toe” OR “clinical check-up” OR “check-up” OR “newborn exam*” OR “physical examination” OR “discharge examination” OR “examination before discharge” OR “clinical examination” OR “physical assessment” OR “physical check-up” OR "clinical assessment” OR (MM "Neonatal Assessment+") OR (MM "Physical Examination+") OR (MH "Developmental Dysplasia of the Hip/DI") OR (MH "Cataract/DI") OR (MH "Heart Defects, Congenital+/DI") OR (MH "Cryptorchidism/DI") OR (MH "Neurologic Examination/MT") OR (MH "Anthropometry/MT") OR (MH "Head Circumference/MT") OR (MH "Body Height/MT") OR (MH "Microcephaly/DI") OR (MH "Jaundice, Neonatal")

Concept string 2 : TI “performance” OR “procedural” OR “psychomotor skills” OR “technical performance” OR “technical skill” OR “evidence-based” OR “gold-standard” OR “gold standard” OR guidance OR guideline* OR standard* OR checklist OR consensus OR recommendation* OR error* OR omission OR mistake* OR (MM "Psychomotor Performance+") OR (MM "Professional Competence+") OR (MM "Clinical Competence+") OR (MH "Professional Practice, Evidence-Based+") OR (MM "Health Care Errors+") OR (MM "Practice Guidelines") OR (MM "Guideline Adherence") OR AB “performance” OR “procedural” OR “psychomotor skills” OR “technical performance” OR “technical skill” OR “evidence-based” OR “gold-standard” OR “gold standard” OR guidance OR guideline* OR standard* OR checklist OR consensus OR recommendation* OR error* OR omission OR mistake* OR (MM "Psychomotor Performance+") OR (MM "Professional Competence+") OR (MM "Clinical Competence+") OR (MH "Professional Practice, Evidence-Based+") OR (MM "Health Care Errors+") OR (MM "Practice Guidelines") OR (MM "Guideline Adherence")

Context-

:

Greene LM, *et al. BMJ Paediatrics Open* 2024; 8:e002286. doi: 10.1136/bmjpo-2023-002286

Appendix IV Data Extraction Template - Coding Framework Headings and Tables

CESoN Procedural Elements Coding

1. Prepare to Examine Neonate

- Timing of examination:
- Introduction by examiner;
- Obtain consent from parent.
- Take a history
    - Maternal
    - Fetal
    - Neonatal
- Equipment Preparation

2. Head-to-Toe Physical Examination & Screening of Neonate

- Appearance,
    - including colour, breathing, behaviour, activity and posture
- Head (including fontanelles), face, nose, mouth, ears, neck and general symmetry of head and facial features
    - visual inspection of palate with wooden tongue depressor and torch
    - digital palpation of hard and soft palate
- Eyes
    - Position, colour of sclera
- Eye **screening**:
    - opacities
    - red reflex
- Neck and clavicles,
- Limbs, hands, feet and digits; assess proportions and symmetry
- Heart: rate
- Heart **screening**:
    - position, rhythm and sounds, murmurs and femoral pulse volume, brachial pulse volume
    - Pulse Oximetry: post -ductal
- Lungs: respiratory effort, rate and lung sounds
- Abdomen: assess shape and palpate to identify any organomegaly; check condition of umbilical cord
- Genitalia and anus: completeness and patency
- Genitalia **screening**: undescended testes in boys
- Spine: inspect and palpate bony structures and check integrity of the skin
- Skin: colour and texture as well as any birthmarks or rashes
- Central nervous system:
    - tone, behaviour, movements and posture; check newborn
    - elicit primitive reflexes
- Hips: symmetry of the limbs,
- Hip **screening**:

  - Barlow manoeuvre
  - Ortolani manoeuvre
- Cry: assess sound
- Measure weight and head circumference of babies in the first week and around 8 weeks, and at other times only if there are concerns. Plot the results on the
- Growth chart
- Measure Length

3. Relay Examination Findings, Baby Health Advice, Document Procedure and Findings.

- Parent/Caregiver Concerns
  - Advice on identifying and managing jaundice
  - If there are concerns about the baby's growth
  - Newborn blood spot screening
  - Newborn hearing screening
- Give parents information about:
  - How to bathe their baby and care for their skin
  - Care of the umbilical stump
  - Feeding
  - Bonding attachment
  - How to recognise if the baby is unwell, and how to seek help
  - Established guidance on safer sleeping
  - Maintaining a smoke-free environment for the baby
  - Vitamin D supplements for babies
  - Immunising the baby
- Advise parents to seek advice from a healthcare professional if they think their baby is unwell, and to contact emergency services (call 999) if they think their baby is seriously ill.
  - Consider giving parents information about the Baby Check scoring system
- Referral to specialist

Study Characteristics Coding

- Study Design: RCT, Quasi -experimental, cohort, diagnostic test accuracy, text & opinion
- Publication status: ongoing, completed
- Publication type: journal article, evidence-based guideline, textbook, national handbook/manual, baby health record book
- Study location:
- **Population** -1 (HCP): numbers, discipline/s.
- Population -2 (Neonates): numbers (if provided), gestation, identified risk factors
- **Concept** – CESoN: Lists the head-to-toe elements or focus on specific element
  - Description of procedural element technique
  - Description of incorrect technique
  - Description of optimal timing

- **Context** – CESoN
  - setting: hospital, primary care, home

- timing: early CESoN (within 6 hours of birth), CESoN 6-24 hours following birth, CESoN greater than 24 hours and less than 72 hours following birth, CESoN greater than 72 hours following birth.

Coding Framework Table 1 – Data Extraction Template (colour coding to aid development of evidence map)

Source	Prepare to Examine Neonate	Head-to-Toe Inspection	Screening - Eyes	Screening - Heart	Screening - Hips	Screening - Testes	Primitive Reflexes	Measure HC and Length	Relay Examination Findings, Baby Health Advice, Document Procedure and Findings.
**Author/s**									
**Year**									
**Geo Location**									
**Type of Source (Design)**<br>**1. Experiment**<br>**2. Cross section/Cohort;**<br>**3. Observational; 4. Educational;**<br>**5. Instructional;**<br>**6. Expert Consensus or Opinion;**									
**Population –**<br>**1. Healthy Term Neonate**<br>**2. At-risk Term neonate**									
**Population –**<br>**1. Qualified Practitioner**<br>**2. Student Learner**									
**Concept Evidence or Theory**									

Greene LM, *et al. BMJ Paediatrics Open* 2024; 8:e002286. doi: 10.1136/bmjpo-2023-002286

**focus**<br>**1. full CESoN;**<br>**2. one organ system only OR one screening only),**									
**Context**<br>**1. CESoN timing,**<br>**2. setting;**									

Coding Framework Table 2 – Mapping CESoN to Types of Evidence

**Source**	**Prepare to Examine Neonate**	**Head-to-Toe Inspection**	**Screening - Eyes**	**Screening - Heart**	**Screening - Hips**	**Screening - Testes**	**Primitive Reflexes**	**Measure HC and Length**	**Relay Examination Findings, Baby Health Advice, Document Procedure and Findings.**
Experimental									
Cross section/Cohort;									
Observational;									
Educational;									
Instructional;									
Expert Consensus or Opinion									

Coding Framework Table 3 – Mapping CESoN Recommendations to Jurisdictions

**Source**	**Prepare to Examine Neonate**	**Head-to-Toe Inspection**	**Screening - Eyes**	**Screening - Heart**	**Screening - Hips**	**Screening - Testes**	**Primitive Reflexes**	**Measure HC and Length**	**Relay Examination Findings, Baby Health Advice, Document Procedure and Findings.**

Ireland									
UK									
USA;									
Canada;									
Australia;									
New Zealand									
Comparison for LMIC: WHO Essentials for P/N Care									

Coding Framework Table 4 – Mapping CESoN Recommendations to descriptions

Source	Prepare to Examine Neonate	Head-to-Toe Inspection	Screening - Eyes	Screening - Heart	Screening - Hips	Screening - Testes	Primitive Reflexes	Measure HC and Length	Relay Examination Findings, Baby Health Advice, Document Procedure and Findings.
Description of technique									
Gold-standard technique									
Omissions									
Errors									

Greene LM, *et al. BMJ Paediatrics Open* 2024; 8:e002286. doi: 10.1136/bmjpo-2023-002286
