# Supplementary material for: Procedural elements of the complete examination and screening of the healthy term neonate: a protocol for a scoping review and evidence map
Source: BMJ Paediatr Open. 2024 Jun 10;8(1):e002286. doi: 10.1136/bmjpo-2023-002286 (PMC11168178; doi:10.1136/bmjpo-2023-002286)
Supplement: Author's manuscript [file bmjpo-2023-002286.draft_revisions.pdf]

BMJ Paediatrics Open

BMJ Paediatrics Open is committed to open peer review. As part of this commitment we make the peer review history of every article we publish publicly available.

When an article is published we post the peer reviewers' comments and the authors' responses online. We also post the versions of the paper that were used during peer review. These are the versions that the peer review comments apply to.

The versions of the paper that follow are the versions that were submitted during the peer review process. They are not the versions of record or the final published versions. They should not be cited or distributed as the published version of this manuscript.

BMJ Paediatrics Open is an open access journal and the full, final, typeset and author-corrected version of record of the manuscript is available on our site with no access controls, subscription charges or pay-per-view fees (http://bmjpaedsopen.bmj.com).

If you have any questions on BMJ Paediatrics Open's open peer review process please email info.bmjpo@bmj.com

BMJ Paediatrics Open

The evidence underpinning the procedural elements of the complete examination and screening of the healthy term neonate: a protocol for a scoping review and evidence map.

Journal:	*BMJ Paediatrics Open*
Manuscript ID	bmjpo-2023-002286
Article Type:	Protocol
Date Submitted by the Author:	15-Sep-2023

Complete List of Authors:	Greene, Liz M; University College Cork, School of Nursing and Midwifery; University College Dublin, School of Nursing, Midwifery and Health Systems<br>O'Connell, Rhona; University College Cork, School of Nursing and Midwifery<br>Murphy, Margaret; University College Cork, School of Nursing and Midwifery<br>Hegarty, Josephine; University College Cork, School of Nursing and Midwifery

Keywords:	Neonatology, Infant

SCHOLARONE™
Manuscripts

I, the Submitting Author has the right to grant and does grant on behalf of all authors of the Work (as defined in the below author licence), an exclusive licence and/or a non-exclusive licence for contributions from authors who are: i) UK Crown employees; ii) where BMJ has agreed a CC-BY licence shall apply, and/or iii) in accordance with the terms applicable for US Federal Government officers or employees acting as part of their official duties; on a worldwide, perpetual, irrevocable, royalty-free basis to BMJ Publishing Group Ltd ("BMJ") its licensees and where the relevant Journal is co-owned by BMJ to the co-owners of the Journal, to publish the Work in this journal and any other BMJ products and to exploit all rights, as set out in our licence.

The Submitting Author accepts and understands that any supply made under these terms is made by BMJ to the Submitting Author unless you are acting as an employee on behalf of your employer or a postgraduate student of an affiliated institution which is paying any applicable article publishing charge ("APC") for Open Access articles. Where the Submitting Author wishes to make the Work available on an Open Access basis (and intends to pay the relevant APC), the terms of reuse of such Open Access shall be governed by a Creative Commons licence – details of these licences and which Creative Commons licence will apply to this Work are set out in our licence referred to above.

Other than as permitted in any relevant BMJ Author's Self Archiving Policies, I confirm this Work has not been accepted for publication elsewhere, is not being considered for publication elsewhere and does not duplicate material already published. I confirm all authors consent to publication of this Work and authorise the granting of this licence.

Confidential: For Review Only

The evidence underpinning the procedural elements of the complete examination and screening of the healthy term neonate: a protocol for a scoping review and evidence map.

Confidential: For Review Only

ABSTRACT

Introduction All newborns undergo a **C**omplete **E**xamination and **S**creening **o**f the **N**eonate (CESoN) to verify the general health and wellbeing of the neonate and to screen for signs and symptoms of illness and significant congenital disorders, typically within 72 hours of birth. For healthy, term gestation neonates, this examination is usually performed by a qualified healthcare practitioner i.e., a midwife or medical doctor, just prior to discharge from the maternity services. As a precursor to modifying and adapting an instrument that measures the quality of performance of the CESoN by healthcare professionals, this review aims to identify, evaluate, synthesise, and map the evidence and theory underpinning current practice and procedural elements of the CESoN.

Methods and analysis This review will be guided by the Joanna Briggs Institute methodology for scoping reviews, and also the recommendations of the Campbell Collaboration for systematic evidence mapping. Based on the research question, the Person, Concept, Context (PCC) framework will be used to develop eligibility criteria for inclusion in the review. Eligible information shall be sourced by searching electronic databases including PubMed, Cumulative Index of Nursing and Allied Health Literature, and Scopus, and the published guidance from expert bodies on newborn examination and screening (e.g., National Institute and Health and Care Excellence, American Academy of Pediatrics, Royal College of Paediatrics and Child Health), and the grey literature. This study will include primary and secondary research papers, evidence-based guidelines, and expert text and opinion published in English from 2013 to September 2023.

Ethics and Dissemination Ethical approval is not required for this scoping review and systematic evidence mapping. The results from this study will be disseminated through peer-reviewed format i.e., conference proceedings, peer-reviewed healthcare journals.

INTRODUCTION

Rationale

The Complete Examination and Screening of the Neonate (CESoN) is a detailed physical examination of the neonate that is performed within a recommended 72 hours following birth. The purpose of this

detailed physical examination in the healthy term gestation neonate is two-fold. First is to verify the general health and wellbeing of the neonate prior to discharge from the maternity service. The second is to screen the baby for signs and symptoms of specific and significant congenital disorders, which if not detected within the recommended timeframe, may result in severe illness [1-5]. The Newborn and Infant Physical Examination (NIPE) programme [5] describes four of the key screening elements (eyes, heart, hips, testes) of the CESoN. A summary of the recommended components of the CESoN is provided (see Appendix I). Optimal performance of the procedural elements of the CESoN is important, particularly the screening elements, given the reality that sub-optimal technique may result in delayed diagnosis and initiation of treatment for progressive congenital disorders [5,6,7]. A 2023 systematic review [8] identified an instrument [9] developed to measure the quality of performance of the complete examination and screening of the neonate by qualified healthcare professionals (HCP).

Objectives

As a precursor to modifying and adapting the identified instrument [9] (which was originally developed and piloted in 2003), this review aims to identify, evaluate, and synthesise the evidence and theory underpinning current practice and procedural elements of this detailed clinical examination. The results of this synthesis and evidence mapping will help guide the future development of standardised practice assessment tools for practitioners of the complete examination and screening of the neonate (CESoN) based on the best available evidence.

The overarching question that this review will attempt to answer is: what are the core and discreet procedural elements of the complete examination and screening of the neonate and their underpinning evidence?

The following sub-questions will structure and focus the data extraction and evidence mapping process:

i. What are the listed discrete elements and core elements outlined within standardised descriptions of the CESoN of the healthy term gestation neonate?

ii. What are the recommended techniques and procedural processes for conducting the various elements of the CESoN of the healthy term gestation neonate?

iii. What are the differences, similarities, variations in suggested practice between standardised descriptions of the CESoN of the healthy term gestation neonate?

iv. What types and quality of evidence underpin recommended best practice of the standardised descriptions of CESoN of the healthy term gestation neonate?

v. What are the omissions, and procedural errors in the conduct of the CESoN of the healthy term gestation neonate?

This scoping review is registered with the Open Science Framework: https:XXXXXX [blinded for peer review process]

METHODS AND ANALYSIS:

Confidential: For Review Only

Leading experts in both scoping review and mapping review methodologies have clarified key differences between these two methodologies [10,11] though it is acknowledged that both types of review have many similarities and overlapping objectives [12].

For the creation of a systematic evidence map, we are referring to the Campbell Collaboration recommendations [13], which cautions researchers that evidence maps "…*summarize what evidence there is, not what the evidence says*".

The review objectives also require us to determine the recommended techniques for conduct of the various procedural elements of the CESoN. Omissions and errors specific to the conduct of the CESoN will also be identified. This goes beyond the above stated evidence mapping review objective of summarising the types of evidence. Therefore, this review must also scope the literature to permit an in-depth analysis of the evidence related to practices and procedural elements specific to the CESoN.

This format of this protocol follows both the author guidelines of the target publication for this protocol [14] and is informed by the recommendations [15] on scoping review protocol items to be reported, and the Preferred Reporting Items for Systematic Review and Meta-Analysis Protocols Checklist (PRISMA-P)[16].

The completed review will be reported using the PRISMA Extension for Scoping Reviews checklist [17] (see Appendix II).

Eligibility Criteria

This review is scoping in nature and seeks to map the evidence underpinning the procedural elements of a multifaceted physical evaluation and screening test performed on all babies within a specific timeframe following birth. Following the recommendations of seminal methodology papers [10,11,15,18] including the scoping review chapter of the JBI Manual for Evidence Synthesis [19], the PCC mnemonic - Participants (P), Concept, (C) and Context (C) is used to define the inclusion criteria for evidence sources. Eligibility criteria must be congruent with the focus and scope of the review question.

Participants (P)

Inclusion criteria for neonates being examined will be a gestation of 37+0 weeks or greater at birth; being born with APGAR score greater than seven at five minutes following birth; being primarily cared for by their parent from birth until their CESoN.

Exclusion criteria for neonates will include neonates born at less than 37 weeks gestation; neonates admitted to a neonatal unit for ongoing care; neonates born with significant congenital disorders or illness diagnosed antenatally or at birth.

Concept (C)

The concept is the recommended best practices of the procedural elements of the complete examination and screening of the healthy term gestation neonate.

Inclusion criteria for concept: complete descriptions of the CESoN or the Newborn Infant Physical Examination (NIPE) will be sought. In addition, description of key elements or groups of steps, for

Confidential: For Review Only

example cardiac screening, developmental dysplasia of the hips (DDH) will be sought, provided they are conducted within the context of the CESoN or the Newborn Infant Physical Examination (NIPE).

Evidence underpinning the complete descriptions of the CESoN or the Newborn Infant Physical Examination (NIPE) will be sought. In addition, evidence underpinning the description of key elements or groups of steps for example cardiac screening, DDH screening will be sought, provided such evidence is collected within the overall context of the CESoN or the Newborn Infant Physical Examination (NIPE).

Context (C)

The context includes the timing and location of the examination. The CESoN may be conducted by a qualified healthcare practitioner in a hospital, primary care, or home setting. The CESoN is a distinct and separate physical examination to the less detailed physical examination of the baby completed within an hour of birth which is typically performed by a midwife [5,20] .

Search Strategy

An initial scoping search using Google Scholar, Google searching, and OneSearch [21] via the University's library search engine was used to iteratively identify relevant keywords and surrogate terms for the core concepts of the search strings using the PCC framework.

The search strategy for the proposed scoping review and evidence mapping will aim to locate both published and unpublished documents using a three-step search strategy. This will involve an initial limited search of both PubMed and CINAHL to identify studies relevant to the topic. Following this initial search, analysis of the text words contained in the titles and abstracts of relevant articles, and the index terms used to describe the articles further informed the development a full search strategy for PubMed (see Appendix III).

A second search will then be conducted across all included databases, incorporating all identified keywords and index terms. The databases to be searched include PubMed, CINAHL (EBSCO), Web of Science Core Collection, Scopus, Maternity and Infant Care (MIDIRS), Cochrane Database of Systematic Reviews, Cochrane Central Register of Controlled trials (CENTRAL), and Joanna Briggs Institute Library.

Thirdly, the reference lists of all identified studies and reports will be screened for additional sources. Sources of unpublished and grey literature, Open Grey, and Google Scholar will also be searched using an abridged version of the original search strings. Similarly, particular websites of relevant expert academic and professional societies will be searched to locate updated national guidance on specific aspects of newborn screening e.g., American Academy of Ophthalmology, Royal College of Paediatrics and Child Health. The key grey literature search terms include newborn and physical examination. In websites or sources where there is a large return of citations, the author will use content relevancy tools, where available, and will screen the first 100 citations for potential inclusion.

A time-limit of 10 years will be applied to assessing eligibility for inclusion.

Types of Evidence Sources

Original research, evidence syntheses, clinical practice guidelines, and guidance published by standard-setting organisations or expert professional bodies. In addition, guidance published within comparable

Confidential: For Review Only

healthcare systems in English-speaking countries, U.K., Ireland, Australia, NZ, Canada, U.S.A., and guidance published in the E.U. in the English language will be included.

Data Charting and Synthesis

Coding and Data Extraction

A key characteristic of evidence mapping is predefining coding categories ahead of the data extraction process [22]. The pre-defined coding categories reflect the information required to address the main objectives of the review question. The characteristics of the studies/papers (e.g., source (author, year, country), primary study design and publication type, participant details, evidence underpinning development of description of the CESoN concept studied, context of CESoN, and key findings relevant to the review questions will be extracted (see Appendix IV).

The data extraction form will be piloted on a sample of three studies by two review authors to assess the feasibility and robustness of the data extraction process. The outcome of the pilot phase will determine whether the data extraction form requires further modifications to comprehensively address the research questions. Any deviations from the above will be outlined in the full scoping review report. Data extraction will be performed by one reviewer (WW) and then checked by a second review author (XX or YY). Other review authors will check random samples of data (20% of total papers included) to ensure high-quality data extraction has been achieved. Any disagreements will be resolved by the involvement of a third review author (ZZ).

The pre-defined coding categories are the 'framework' to classify the data for the evidence synthesis. As our review question and objectives do not align with the frameworks applied to standard evidence mapping reviews e.g., 3ie gap maps, Sightsaver 3 EGMs, we will develop a framework based on the procedural elements of the CESoN. These elements are an amalgamation drawn from three institutions – the American Academy of Pediatrics, the National Institute of Health and Care Excellence, and Public Health England. These internationally recognised expert institutions have published recommendations on the CESoN [1,4,5]. Initially the procedural elements and associated omissions and errors extracted from the included papers are mapped to the three broad themes:

1. Preparation of the neonate for the examination
2. Details for the systematic process and procedural steps for the physical examination and screening
3. Documentation, sharing details of the findings of the examination, providing advice and information

Comparisons are drawn across included papers in terms of numbers of steps, sequencing of steps and errors identified. Once all data is extracted, descriptions of each step are compared and condensed. The aim for each step of the process is to iteratively create a summative, salient, and essence-capturing step with a clear beginning and end to a step outlined, based upon the comparison of descriptions provided. Errors associated with each step are also summarised.

In order to address certain research sub-questions (i, iv), the data extraction coding framework will also include a mapping of the evidence underpinning recommended best practice for discrete steps or groups of steps relating to discrete assessments conducted as part of the overall CESoN.

Confidential: For Review Only

A narrative summary will accompany the tabulated and/or charted results and will describe how the results relate to the reviews objective and question/s.

Quality Assessment

For the traditional scoping review, an assessment of the strength of the body of evidence using the Grading of Recommendations, Assessment, Development and Evaluation (GRADE) tool is not ordinarily conducted [15].

Seminal papers on scoping review and mapping review methodologies state that critical appraisal of included studies is optional [10,11]. However, other notable papers on mapping review methodology [13,22] recommend critical appraisal of selected included studies to increase confidence in the findings of the evidence synthesis e.g., systematic reviews, primary studies.

For papers which describe the complete CESoN or NIPE the review authors will describe the processes which underpinned the development of the description or guidance outlining the procedural elements and steps of the CESoN.

The quality of evidence underpinning the elements of CESoN will be assessed, where possible, using quality assessment tools appropriate to each evidence source i.e., primary research, secondary research sources. For example, the quality of any systematic reviews of certain elements of the CESoN (e.g., physical examination and screening of the hips for DDH) will be assessed using the JBI critical appraisal tool for systematic reviews [23] . Where a clinical practice guideline is identified, it will be assessed for methodological quality using appraisal tools such as the CASP checklists and AGREE II checklist [24]. For expert text and opinion sources, the JBI critical appraisal tool will be used [25].

ETHICS AND DISSEMINATION

Ethical approval is not required for this scoping review and systematic evidence mapping. The results from this study will be disseminated through peer-reviewed processes for example conference proceedings, peer-reviewed healthcare journals. The final review will also be included with the doctoral thesis of the lead author (WW) and made publicly available on the university's Open Access institutional repository.

Authors' contributions:

WW, XX, YY, and ZZ [pseudonyms for peer review process] involved in conception & design of review, acquisition & analysis of data, interpretation of findings, drafting and reviewing manuscripts.

Funding statement: 'This research received no specific grant from any funding agency in the public, commercial or not-for-profit sectors'. The doctoral studies of the lead author (WW), of which this work is in part-fulfilment, are funded by their employer (University B).

Competing interests statement The authors of this paper have no competing interests to declare.

What is already known on this topic

Confidential: For Review Only

The Complete Examination and Screening of the Neonate (CESoN) is an important, universal baseline assessment of newborn health and wellbeing. Despite its importance, there is a paucity of research on many of the psychomotor and assessment skills utilised by clinicians to conduct the CESoN.

What this study hopes to add

This study hopes to provide clarity on the both the sources and levels of evidence underpinning recommendations for optimal performance of the CESoN. This scoping review is part of an evidence mapping process to inform modifications that may be needed to update and pilot an instrument that measures clinician performance of the CESoN.

How this study might affect research, practice or policy

This study may positively affect practice by providing clinicians and academics with an evidence map that illustrates the strength of evidence underpinning the identified performance elements of the CESoN. As the ultimate goal of the CESoN is to protect neonatal health by detecting early signs and symptoms that may indicate illness or congenital disorders, it is vital that the examination and screening techniques are aligned to the latest and best level-evidence available.

Patient and Public Involvement Statement:

Patients or the public will not be involved in the design or conduct of our research. We will report and disseminate our research findings in the public domain.

Confidential: For Review Only

Keywords

Newborn; Physical Examination; Psychomotor Performance; Evidence-Based Practice.

Acknowledgements

Contributions of individuals, or institutions, who have not already been listed as co-authors will be provided. This review is to contribute towards a doctoral degree award for author WW [blinded for peer review process].

Confidential: For Review Only

References

1. Benitz, W.E. et al. 2015. Hospital stay for healthy term newborn infants. *Pediatrics (Evanston)* 135(5) 948-953. DOI: 10.1542/peds.2015-0699
2. Health Service Executive (HSE). The Newborn Clinical Examination Handbook [online]. HSE. 2018. https://www.hse.ie/eng/about/who/healthwellbeing/our-priority-programmes/child-health-and-wellbeing/neonate%20exam.pdf (accessed 01 July 2023).
3. Lemyre B, Jefferies AL, O'Flaherty P. Facilitating discharge from hospital of the healthy term infant. Paediatrics & child health. 2018;23(8):515-22. DOI: 10.1093/pch/pxy127
4. National Institute of Health and Care Excellence (NICE) Postnatal Care NICE guideline NG194. *1.3 Postnatal care of the baby*. NICE. 2021. 18-21. https://www.nice.org.uk/guidance/ng194 (accessed 01 July 2023).
5. Public Health England (PHE) Newborn and infant physical examination (NIPE) screening programme handbook. *1. NIPE screening examination*. PHE. 2021. https://www.gov.uk/government/publications/newborn-and-infant-physical-examination-programme-handbook/newborn-and-infant-physical-examination-screening-programme-handbook#training-and-competency (accessed 01 July 2023).
6. Paton RW. Screening in Developmental Dysplasia of the Hip (DDH). The surgeon (Edinburgh). 2017;15(5):290-6. DOI: 10.1016/j.surge.2017.05.002
7. Martin GR, Ewer AK, Gaviglio A, Hom LA, Saarinen A, Sontag M, et al. Updated Strategies for Pulse Oximetry Screening for Critical Congenital Heart Disease. Pediatrics. DOI: 10.1542/peds.2019-16502020;146(1):e20191650.
8. BLINDED for peer review process
9. Townsend, J. et al. 2004. Routine examination of the newborn: the EMREN study. Evaluation of an extension of the midwife role including a randomised controlled trial of appropriately trained midwives and paediatric senior house officers - Introduction. *Health technology assessment (Winchester, England)* 8(14) 1. DOI: 10.3310/hta8140
10. Campbell F, Tricco AC, Munn Z, Pollock D, Saran A, Sutton A, et al. Mapping reviews, scoping reviews, and evidence and gap maps (EGMs): the same but different- the "Big Picture" review family. Syst Rev. 2023;12(1):45. DOI: 10.1186/s13643-023-02178-5
11. Khalil H, Tricco AC. Differentiating between mapping reviews and scoping reviews in the evidence synthesis ecosystem. Journal of clinical epidemiology. 2022;149:175-82. DOI: 10.1016/j.jclinepi.2022.05.012
12. Chen Y-F, Takwoingi Y, Grove A. Mapping review could be seen as a subtype of scoping review and differentiating between the action of mapping evidence and presentation of evidence as maps may be helpful: response to Khalil et al. Journal of clinical epidemiology. 2023;157:110-1. DOI: 10.1016/j.jclinepi.2023.02.017
13. Saran A, White H. Evidence and gap maps: a comparison of different approaches. Campbell Systematic Reviews. 2018;14(1):1-38. https://doi.org/10.4073/cmdp.2018.2
14. BMJ Paediatrics Open. Submission Guidelines online: BMJ Publishing & RCPCH; 2023. https://bmjpaedsopen.bmj.com/pages/authors/ (accessed 15 August 2023).
15. Peters MDJ, Godfrey C, McInerney P, et alBest practice guidance and reporting items for the development of scoping review protocols. JBI Evidence Synthesis. 2022;20(4):953-68. DOI: 10.11124/jbies-21-00242
16. Moher D, Shamseer L, Clarke M, et alPreferred reporting items for systematic review and meta-analysis protocols (PRISMA-P) 2015 statement. Systematic Reviews. 2015;4(1):1. DOI: 10.1186/2046-4053-4-1

Confidential: For Review Only

17. Tricco A.C. LE, Zarin W, O'Brien K.K, et alPRISMA Extension for Scoping Reviews (PRISMA-ScR): Checklist and Explanation. Annals of Internal Medicine. 2018;169(7):467-73. https://doi.org/10.7326/M18-0850
18. Khalil H, Peters MDJ, Tricco AC, Pollock D, Alexander L, McInerney P, et al. Conducting high quality scoping reviews-challenges and solutions. Journal of Clinical Epidemiology. 2021;130:156-60. https://doi.org/10.1016/j.jclinepi.2020.10.009
19. Aromataris E, Munn Z (Editors). *JBI Manual for Evidence Synthesis.* JBI, 2020. https://synthesismanual.jbi.global. https://doi.org/10.46658/JBIMES-20-01 (accessed 06 July 2023).
20. W.H.O. Pregnancy, childbirth, postpartum and newborn care: A guide for essential practice (3rd edition) (PCPNC). 2015. Newborn Care. Geneva, World Health Organisation. 184. https://www.ncbi.nlm.nih.gov/books/NBK326678/ (accessed 01 July 2023).
21. UCC Library (2023) What is OneSearch and how do I use it? https://askus.booleweb.ucc.ie/research/faq/190571#:~:text=OneSearch%20is%20a%20single%2Dsearch,on%20the%20UCC%20Library%20homepage (accessed 15 August 2023).
22. White, H. et al. 2020. Guidance for producing a Campbell evidence and gap map. *Campbell Syst Rev* 16(4) e1125. DOI: 10.1002/cl2.1125
23. Aromataris, E. et al. 2015. Summarizing systematic reviews: methodological development, conduct and reporting of an umbrella review approach. *JBI Evidence Implementation* 13(3) 132-140. DOI: 10.1097/xeb.0000000000000055
24. Brouwers, M.C. et al. 2010. AGREE II: advancing guideline development, reporting and evaluation in health care. *Canadian Medical Association journal (CMAJ)* 182(18) E839-842. DOI: 10.1503/cmaj.090449
25. McArthur A, K.J., Yan H, Florescu S. 2020. JBI Critical Appraisal Checklist for Text and Opinion Papers. *In* M. Z. Aromataris E ed. *JBI Manual for Evidence Synthesis*. Joanna Briggs Institute. https://doi.org/10.46658/JBIMES-20-05

Confidential: For Review Only

Confidential: For Review Only

Supplemental File ScEvMap_CESoN Procedure Protocol_15092023

Contents

Appendix I The Components of the Complete Examination and Screening of the Healthy Term Neonate (CESoN) 1

Appendix II Reporting Guidelines PRISMA ScR Checklist (17) 4

Appendix III: Search strategy for both PubMed and CINAHL 6

Appendix IV Data Extraction Template - Coding Framework Headings and Tables 8

CESoN Procedural Elements Coding 8

Study Characteristics Coding 9

Confidential: For Review Only

Appendix I The Components of the Complete Examination and Screening of the Healthy Term Neonate (CESoN)

CESoN	American Academy of Pediatrics [1]	National Institute of Health and Care Excellence (NICE) [4]	Public Health England (PHE) [5]
**Prepare to Examine Neonate**	Clinical course reveals no abnormalities that require continued hospitalization.<br><br>Review baby vital signs for preceding 12 hours.<br><br>Risk factors for sepsis?<br><br>Assess quality of mother-infant attachment and details of infant behaviour	Review the history.<br>Ask parents if they have any concerns about their baby's general wellbeing… or development.	Ask: mother's medical and recent obstetric history, including any medication baby's family history baby's immediate postnatal health.<br>Check risk factors for eye disorders, CHD, DDH, and undescended testes/testis.
	has completed at least 2 successful feedings	Ask parents if they have any concerns about their baby's …feeding	
	infant has urinated regularly and passed at least 1 stool	Meconium passed within 24 hours of birth	
**Head-to-Toe Physical Examination and Screening**	Physical examination reveals no abnormalities that require continued hospitalization.<br><br>Check for jaundice.	Appearance, including colour, breathing, behaviour, activity and posture	
		Head (including fontanelles), face, nose, mouth (including palate), ears, neck and general symmetry of head and facial features	
		Eyes: opacities, red reflex and colour of sclera	Asses position, structure, and red reflex.

		Neck and clavicles, limbs, hands, feet and digits; assess proportions and symmetry	
		heart: position, heart rate, rhythm and sounds, murmurs and femoral pulse volume	Observation of tone, colour, size and shape of chest, respiratory rate and effort.<br>Palpation of femoral and brachial pulse, capillary refill time, chest, liver.<br>Auscultation of pulmonic, aortic, tricuspid, mitral, and midscapulae/coarctation areas.
		Lungs: respiratory effort, rate and lung sounds	
		abdomen: assess shape and palpate to identify any organomegaly; check condition of umbilical cord	
	No evidence of excessive bleeding at the circumcision site at least 2 hours.	Genitalia and anus: completeness and patency and undescended testes in boys	(In males) Observe the scrotum for symmetry, size and colour. Palpation of scrotal sac (+inguinal canal and perineum if testis not located in scrotal sac).
		Spine: inspect and palpate bony structures and check integrity of the skin	
		Skin: colour and texture as well as any birthmarks or rashes	
		Central nervous system: tone, behaviour, movements and posture; check newborn reflexes only if concerned	
		Hips: symmetry of the limbs, Barlow and Ortolani's manoeuvres	Observation of leg length, symmetry. Manipulation of each hip with Barlow and Ortolani test manoeuvres.
		Cry: assess sound.	
	weigh the infant	Measure weight and head circumference of babies in the first week	
**Relay Examination Findings, Baby Health Advice, Document Procedure and Findings.**	Appropriate jaundice management and/or follow-up plans have been instituted	Advice on identifying and managing jaundice	

Confidential: For Review Only

	Newborn metabolic and hearing screenings have been completed per hospital protocol	Carry out/check bloodspot and hearing screening performed	
	Newborn pulse oximetry screening has been completed per hospital protocol		
	training and information to mother about: breastfeeding, baby urine and stool patterns, baby care including cord, use of thermometer, signs symptoms of unwell baby, Immunisation, infant safety including car seat usage, prevention of SIDS, and hand hygiene	Give parents information about: baby care including cord, feeding, bonding, signs symptoms of unwell baby, prevention of SIDS, Vitamin D supplementation, Immunisation	

Confidential: For Review Only

Appendix II Reporting Guidelines PRISMA ScR Checklist (17)

SECTION	ITEM	PRISMA-ScR CHECKLIST ITEM	REPORTED ON PAGE #
**TITLE**			
Title	1	Identify the report as a scoping review.	1
**ABSTRACT**			
Structured summary	2	Provide a structured summary that includes (as applicable): background, objectives, eligibility criteria, sources of evidence, charting methods, results, and conclusions that relate to the review questions and objectives.	1
**INTRODUCTION**			
Rationale	3	Describe the rationale for the review in the context of what is already known. Explain why the review questions/objectives lend themselves to a scoping review approach.	2
Objectives	4	Provide an explicit statement of the questions and objectives being addressed with reference to their key elements (e.g., population or participants, concepts, and context) or other relevant key elements used to conceptualize the review questions and/or objectives.	2
**METHODS**			
Protocol and registration	5	Indicate whether a review protocol exists; state if and where it can be accessed (e.g., a Web address); and if available, provide registration information, including the registration number.	2
Eligibility criteria	6	Specify characteristics of the sources of evidence used as eligibility criteria (e.g., years considered, language, and publication status), and provide a rationale.	3
Information sources*	7	Describe all information sources in the search (e.g., databases with dates of coverage and contact with authors to identify additional sources), as well as the date the most recent search was executed.	3,4
Search	8	Present the full electronic search strategy for at least 1 database, including any limits used, such that it could be repeated.	4,5
Selection of sources of evidence†	9	State the process for selecting sources of evidence (i.e., screening and eligibility) included in the scoping review.	5
Data charting process‡	10	Describe the methods of charting data from the included sources of evidence (e.g., calibrated forms or forms that have been tested by the team before their use, and whether data charting was done independently or in duplicate) and any processes for obtaining and confirming data from investigators.	5
Data items	11	List and define all variables for which data were sought and any assumptions and simplifications made.	5,6
Critical appraisal of individual sources of evidence§	12	If done, provide a rationale for conducting a critical appraisal of included sources of evidence; describe the methods used and how this information was used in any data synthesis (if appropriate).	6
Synthesis of results	13	Describe the methods of handling and summarizing the data that were charted.	Click here to enter text.
**RESULTS**			
Selection of sources of evidence	14	Give numbers of sources of evidence screened, assessed for eligibility, and included in the review, with reasons for exclusions at each stage, ideally using a flow diagram.	Click here to enter text.

Confidential: For Review Only

SECTION	ITEM	PRISMA-ScR CHECKLIST ITEM	REPORTED ON PAGE #
Characteristics of sources of evidence	15	For each source of evidence, present characteristics for which data were charted and provide the citations.	Click here to enter text.
Critical appraisal within sources of evidence	16	If done, present data on critical appraisal of included sources of evidence (see item 12).	Click here to enter text.
Results of individual sources of evidence	17	For each included source of evidence, present the relevant data that were charted that relate to the review questions and objectives.	Click here to enter text.
Synthesis of results	18	Summarize and/or present the charting results as they relate to the review questions and objectives.	Click here to enter text.
**DISCUSSION**			
Summary of evidence	19	Summarize the main results (including an overview of concepts, themes, and types of evidence available), link to the review questions and objectives, and consider the relevance to key groups.	Click here to enter text.
Limitations	20	Discuss the limitations of the scoping review process.	Click here to enter text.
Conclusions	21	Provide a general interpretation of the results with respect to the review questions and objectives, as well as potential implications and/or next steps.	Click here to enter text.
**FUNDING**			
Funding	22	Describe sources of funding for the included sources of evidence, as well as sources of funding for the scoping review. Describe the role of the funders of the scoping review.	7

Confidential: For Review Only

Appendix III: Search strategy for both PubMed and CINAHL

Limits: *Years: 2013-2023; English language; Humans*

PubMed 2023 earch strings keywords (abstract or title) and MeSH SHs:

P: “baby” OR “babies” OR “newborn” OR “newborns” OR “new-born” OR “new-borns” OR “neonate” OR “neonates” OR “neonatal” OR “infant” OR “infants” OR (MM "Infant, Newborn+") OR "infant, newborn"[MeSH Terms] OR "baby"[Title/Abstract] OR "babies"[Title/Abstract] OR "newborn"[Title/Abstract] OR "newborns"[Title/Abstract] OR "new-born"[Title/Abstract] OR "new-borns"[Title/Abstract] OR "neonate"[Title/Abstract] OR "neonates"[Title/Abstract] OR "neonatal"[Title/Abstract] OR "infant"[Title/Abstract] OR "infants"[Title/Abstract]

Concept string 1: "newborn infant physical examination" OR "NIPE" OR “head-to-toe” OR “head to toe” OR “clinical check-up” OR “check-up” OR “newborn exam*” OR “physical examination” OR “discharge examination” OR “examination before discharge” OR “clinical examination” OR “physical assessment” OR “physical check-up” OR "clinical assessment” OR (MM "Physical Examination+") OR ("Physical Examination/instrumentation"[Majr] OR "Physical Examination/methods"[Majr] OR "Physical Examination/standards"[Majr]) OR "newborn infant physical examination"[Title/Abstract] OR "NIPE"[Title/Abstract] OR "head-to-toe"[Title/Abstract] OR "check-up"[Title/Abstract] OR "checkup"[Title/Abstract] OR "newborn examination"[Title/Abstract] OR "physical examination"[Title/Abstract] OR "discharge examination"[Title/Abstract] OR "detailed physical"[Title/Abstract] OR "clinical examination"[Title/Abstract] OR "physical assessment"[Title/Abstract] OR "physical check-up"[Title/Abstract] OR "clinical assessment"[Title/Abstract] OR "developmental dysplasia of the hip/diagnosis"[MeSH Major Topic] OR "cataract/congenital"[MeSH Major Topic] OR "heart defects, congenital/diagnosis"[MeSH Major Topic] OR "cryptorchidism/diagnosis"[MeSH Major Topic] OR "neurologic examination/methods"[MeSH Major Topic] OR "Body Height"[MeSH Major Topic] OR "megalencephaly/diagnosis"[MeSH Major Topic] OR "microcephaly/diagnosis"[MeSH Major Topic] OR "Jaundice, Neonatal/diagnosis"[Majr]

Concept string 2: “performance” OR “procedural” OR “psychomotor skills” OR “technical performance” OR “technical skill” OR “evidence-based” OR “gold-standard” OR “gold standard” OR guidance OR guideline* OR standard* OR checklist OR consensus OR recommendation* OR error* OR omission OR mistake* OR "Psychomotor Performance"[Mesh] OR “Clinical Competence"[Mesh] OR "Evidence-Based Practice"[Mesh] OR "Psychomotor Performance"[MeSH Terms] OR "Clinical Competence"[MeSH Terms] OR "Evidence-Based Practice"[MeSH Terms] OR "Medical Errors"[MeSH Terms] OR "Guideline Adherence"[MeSH Terms] OR "Professional Competence/standards"[Mesh] OR "Standard of Care"[MeSH Terms] OR "performance"[Title/Abstract] OR "procedural"[Title/Abstract] OR "psychomotor skill*"[Title/Abstract] OR "technical performance"[Title/Abstract] OR "technical skill*"[Title/Abstract] OR "technique*"[Title/Abstract] OR "step*"[Title/Abstract] OR "evidence-based"[Title/Abstract] OR "gold-standard*"[Title/Abstract] OR "gold standard*"[Title/Abstract] OR guidance[Title/Abstract] OR guideline*[Title/Abstract] OR standard*[Title/Abstract] OR checklist*[Title/Abstract] OR consensus[Title/Abstract] OR recommendation*[Title/Abstract] OR error*[Title/Abstract] OR omission*[Title/Abstract] OR mistake*[Title/Abstract]

Context-

Confidential: For Review Only

CINAHL 2023 search strings keywords (abstract or title) and MeSH SHs:

Person: TI “baby” OR “babies” OR “newborn” OR “newborns” OR “new-born” OR “new-borns” OR “neonate” OR “neonates” OR “neonatal” OR “infant” OR “infants” OR (MM "Infant, Newborn+") OR AB “baby” OR “babies” OR “newborn” OR “newborns” OR “new-born” OR “new-borns” OR “neonate” OR “neonates” OR “neonatal” OR “infant” OR “infants” OR (MM "Infant, Newborn+")

Concept string 1 : TI "newborn infant physical examination" OR "NIPE" OR “head-to-toe” OR “head to toe” OR “clinical check-up” OR “check-up” OR “newborn exam*” OR “physical examination” OR “discharge examination” OR “examination before discharge” OR “clinical examination” OR “physical assessment” OR “physical check-up” OR "clinical assessment” OR (MM "Neonatal Assessment+") OR (MM "Physical Examination+") OR (MH "Developmental Dysplasia of the Hip/DI") OR (MH "Cataract/DI") OR (MH "Heart Defects, Congenital+/DI") OR (MH "Cryptorchidism/DI") OR (MH "Neurologic Examination/MT") OR (MH "Anthropometry/MT") OR (MH "Head Circumference/MT") OR (MH "Body Height/MT") OR (MH "Microcephaly/DI") OR (MH "Jaundice, Neonatal") OR AB "newborn infant physical examination" OR "NIPE" OR “head-to-toe” OR “head to toe” OR “clinical check-up” OR “check-up” OR “newborn exam*” OR “physical examination” OR “discharge examination” OR “examination before discharge” OR “clinical examination” OR “physical assessment” OR “physical check-up” OR "clinical assessment” OR (MM "Neonatal Assessment+") OR (MM "Physical Examination+") OR (MH "Developmental Dysplasia of the Hip/DI") OR (MH "Cataract/DI") OR (MH "Heart Defects, Congenital+/DI") OR (MH "Cryptorchidism/DI") OR (MH "Neurologic Examination/MT") OR (MH "Anthropometry/MT") OR (MH "Head Circumference/MT") OR (MH "Body Height/MT") OR (MH "Microcephaly/DI") OR (MH "Jaundice, Neonatal")

Concept string 2 : TI “performance” OR “procedural” OR “psychomotor skills” OR “technical performance” OR “technical skill” OR “evidence-based” OR “gold-standard” OR “gold standard” OR guidance OR guideline* OR standard* OR checklist OR consensus OR recommendation* OR error* OR omission OR mistake* OR (MM "Psychomotor Performance+") OR (MM "Professional Competence+") OR (MM "Clinical Competence+") OR (MH "Professional Practice, Evidence-Based+") OR (MM "Health Care Errors+") OR (MM "Practice Guidelines") OR (MM "Guideline Adherence") OR AB “performance” OR “procedural” OR “psychomotor skills” OR “technical performance” OR “technical skill” OR “evidence-based” OR “gold-standard” OR “gold standard” OR guidance OR guideline* OR standard* OR checklist OR consensus OR recommendation* OR error* OR omission OR mistake* OR (MM "Psychomotor Performance+") OR (MM "Professional Competence+") OR (MM "Clinical Competence+") OR (MH "Professional Practice, Evidence-Based+") OR (MM "Health Care Errors+") OR (MM "Practice Guidelines") OR (MM "Guideline Adherence")

Context-

:

7

Confidential: For Review Only

Appendix IV Data Extraction Template- Coding Framework Headings and Tables

CESoN Procedural Elements Coding

1. Prepare to Examine Neonate

- Timing of examination:
- Introduction by examiner;
- Obtain consent from parent.
- Take a History
  - Maternal
  - Fetal
  - Neonatal
- Equipment Preparation

2. Head-to-Toe Physical Examination & Screening of Neonate

- Appearance,
  - including colour, breathing, behaviour, activity and posture
- Head (including fontanelles), face, nose, mouth, ears, neck and general symmetry of head and facial features
  - visual inspection of palate with wooden tongue depressor and torch
  - digital palpation of hard and soft palate
- Eyes
  - Position, colour of sclera
- Eye **screening**:
  - opacities
  - red reflex
- Neck and clavicles,
- Limbs, hands, feet and digits; assess proportions and symmetry
- Heart: rate
- Heart **screening**:
  - position, rhythm and sounds, murmurs and femoral pulse volume, brachial pulse volume
  - Pulse Oximetry: post -ductal
- Lungs: respiratory effort, rate and lung sounds
- Abdomen: assess shape and palpate to identify any organomegaly; check condition of umbilical cord
- Genitalia and anus: completeness and patency
- Genitalia **screening**: undescended testes in boys
- Spine: inspect and palpate bony structures and check integrity of the skin
- Skin: colour and texture as well as any birthmarks or rashes
- Central nervous system:
  - tone, behaviour, movements and posture; check newborn
  - elicit primitive reflexes
- Hips: symmetry of the limbs,
- Hip **screening**:

Confidential: For Review Only

    - Barlow manoeuvre
    - Ortolani manoeuvre
- Cry: assess sound
- Measure weight and head circumference of babies in the first week and around 8 weeks, and at other times only if there are concerns. Plot the results on the
- Growth chart
- Measure Length

3. Relay Examination Findings, Baby Health Advice, Document Procedure and Findings.

- Parent/Caregiver Concerns
    - Advice on identifying and managing jaundice
    - If there are concerns about the baby's growth
    - Newborn blood spot screening
    - Newborn hearing screening
- Give parents information about:
    - How to bathe their baby and care for their skin
    - Care of the umbilical stump
    - Feeding
    - Bonding attachment
    - How to recognise if the baby is unwell, and how to seek help
    - Established guidance on safer sleeping
    - Maintaining a smoke-free environment for the baby
    - Vitamin D supplements for babies
    - Immunising the baby
- Advise parents to seek advice from a healthcare professional if they think their baby is unwell, and to contact emergency services (call 999) if they think their baby is seriously ill.
    - Consider giving parents information about the Baby Check scoring system
- Referral to specialist

Study Characteristics Coding

- Study Design: RCT, Quasi -experimental, cohort, diagnostic test accuracy, text & opinion
- Publication status: ongoing, completed
- Publication type: journal article, evidence-based guideline, textbook, national handbook/manual, baby health record book
- Study location:
- **Population** -1 (HCP): numbers, discipline/s.
- Population -2 (Neonates): numbers (if provided), gestation, identified risk factors
- **Concept** – CESoN: Lists the head-to-toe elements or focus on specific element
    - Description of procedural element technique
    - Description of incorrect technique
    - Description of optimal timing

- **Context** – CESoN
    - setting: hospital, primary care, home

Confidential: For Review Only

- timing: early CESoN (within 6 hours of birth), CESoN 6-24 hours following birth, CESoN greater than 24 hours and less than 72 hours following birth, CESoN greater than 72 hours following birth.

Confidential: For Review Only

Coding Framework Table 1 – **Data Extraction Template** (colour coding to aid development of evidence map)

Source	Prepare to Examine Neonate	Head-to-Toe Inspection	Screening - Eyes	Screening - Heart	Screening - Hips	Screening - Testes	Primitive Reflexes	Measure HC and Length	Relay Examination Findings, Baby Health Advice, Document Procedure and Findings.
**Author/s**									
**Year**									
**Geo Location**									
**Type of Source (Design)**<br>**1. Experiment**<br>**2. Cross section/Cohort;**<br>**3. Observational; 4. Educational;**<br>**5. Instructional;**<br>**6. Expert Consensus or Opinion;**									
**Population –**<br>**1. Healthy Term Neonate**<br>**2. At-risk Term neonate**									
**Population –**<br>**1. Qualified Practitioner**<br>**2. Student Learner**									
**Concept**									

**Evidence or Theory focus**<br>**1. full CESoN;**<br>**2. one organ system only OR one screening only),**									
**Context**<br>**1. CESoN timing,**<br>**2. setting;**									

Coding Framework Table 2 – Mapping CESoN to Types of Evidence

**Source**	**Prepare to Examine Neonate**	**Head-to-Toe Inspection**	**Screening - Eyes**	**Screening - Heart**	**Screening - Hips**	**Screening - Testes**	**Primitive Reflexes**	**Measure HC and Length**	**Relay Examination Findings, Baby Health Advice, Document Procedure and Findings.**
Experimental									
Cross section/Cohort;									
Observational;									
Educational;									
Instructional;									
Expert Consensus or Opinion									

Coding Framework Table 3 – Mapping CESoN Recommendations to Jurisdictions

**Source**	**Prepare to Examine Neonate**	**Head-to-Toe Inspection**	**Screening - Eyes**	**Screening - Heart**	**Screening - Hips**	**Screening - Testes**	**Primitive Reflexes**	**Measure HC and Length**	**Relay Examination Findings, Baby Health Advice, Document**

									Procedure and Findings.
Ireland									
UK									
USA;									
Canada;									
Australia;									
New Zealand									
Comparison for LMIC: WHO Essentials for P/N Care									

Coding Framework Table 4 – Mapping CESoN Recommendations to descriptions

**Source**	**Prepare to Examine Neonate**	**Head-to-Toe Inspection**	**Screening - Eyes**	**Screening - Heart**	**Screening - Hips**	**Screening - Testes**	**Primitive Reflexes**	**Measure HC and Length**	**Relay Examination Findings, Baby Health Advice, Document Procedure and Findings.**
Description of technique									
Gold-standard technique									
Omissions									
Errors									

BMJ Paediatrics Open

The procedural elements of the complete examination and screening of the healthy term neonate: a protocol for a scoping review and evidence map.

Journal:	*BMJ Paediatrics Open*
Manuscript ID	bmjpo-2023-002286.R1
Article Type:	Protocol
Date Submitted by the Author:	29-Apr-2024
Complete List of Authors:	Greene, Liz M; University College Cork, School of Nursing and Midwifery; University College Dublin, School of Nursing, Midwifery and Health Systems<br>O'Connell, Rhona; University College Cork, School of Nursing and Midwifery<br>Murphy, Margaret; University College Cork, School of Nursing and Midwifery<br>Hegarty, Josephine; University College Cork, School of Nursing and Midwifery
Keywords:	Neonatology, Infant

SCHOLARONE™
Manuscripts

I, the Submitting Author has the right to grant and does grant on behalf of all authors of the Work (as defined in the below author licence), an exclusive licence and/or a non-exclusive licence for contributions from authors who are: i) UK Crown employees; ii) where BMJ has agreed a CC-BY licence shall apply, and/or iii) in accordance with the terms applicable for US Federal Government officers or employees acting as part of their official duties; on a worldwide, perpetual, irrevocable, royalty-free basis to BMJ Publishing Group Ltd ("BMJ") its licensees and where the relevant Journal is co-owned by BMJ to the co-owners of the Journal, to publish the Work in this journal and any other BMJ products and to exploit all rights, as set out in our licence.

The Submitting Author accepts and understands that any supply made under these terms is made by BMJ to the Submitting Author unless you are acting as an employee on behalf of your employer or a postgraduate student of an affiliated institution which is paying any applicable article publishing charge ("APC") for Open Access articles. Where the Submitting Author wishes to make the Work available on an Open Access basis (and intends to pay the relevant APC), the terms of reuse of such Open Access shall be governed by a Creative Commons licence – details of these licences and which Creative Commons licence will apply to this Work are set out in our licence referred to above.

Other than as permitted in any relevant BMJ Author's Self Archiving Policies, I confirm this Work has not been accepted for publication elsewhere, is not being considered for publication elsewhere and does not duplicate material already published. I confirm all authors consent to publication of this Work and authorise the granting of this licence.

Confidential: For Review Only

Supplemental File ScEvMap_CESoN Procedure Protocol

Contents

Appendix I The Components of the Complete Examination and Screening of the Healthy Term Neonate (CESoN)1
Appendix II Reporting Guidelines PRISMA ScR Checklist (17) 4
Appendix III: Search strategy for both PubMed and CINAHL 6
Appendix IV Data Extraction Template - Coding Framework Headings and Tables 8
CESoN Procedural Elements Coding 8
Study Characteristics Coding 9

Appendix I The Procedural Elements of the Complete Examination and Screening of the Healthy Term Neonate (CESoN)

CESoN	Public Health England (PHE) [1]	National Institute of Health and Care Excellence (NICE) [2]	American Academy of Pediatrics [3]
**Prepare to Examine Neonate**	Ask: mother's medical and recent obstetric history, including any medication	Review the history.<br><br>Meconium passed within 24 hours of birth	Clinical course reveals no abnormalities that require continued hospitalization.<br><br>Review baby vital signs for preceding 12 hours.<br><br>Risk factors for sepsis?<br><br>Assess quality of mother-infant attachment and details of infant behaviour
	Check risk factors for eye disorders, CHD, DDH, and undescended testes/testis	Ask parents if they have any concerns about their baby's general wellbeing… or development.	has completed at least 2 successful feedings
	baby's family history	Ask parents if they have any concerns about their baby's …feeding	infant has urinated regularly and passed at least 1 stool
**Head-to-Toe Physical Examination and Screening**	baby's immediate postnatal health	Appearance, including colour, breathing, behaviour, activity and posture<br><br>Cry: assess sound.	Physical examination reveals no abnormalities that require continued hospitalization.<br><br>Check for jaundice.
		Head (including fontanelles), face, nose, mouth (including palate), ears, neck and general symmetry of head and facial features	
	Eyes: Asses position, structure, and red reflex.	Eyes: opacities, red reflex and colour of sclera	
		Neck and clavicles, limbs, hands, feet and digits; assess proportions and symmetry	

1

Commented [A1]: No.4 changed from 'components' to 'procedural elements' to clarify the focus of this scoping review.

Commented [A2]: No.7 - Thank you for this observation. Appendix 1 sets out the procedural elements of the routine CESoN, as per the guidelines of three internationally recognised standard-setting organisations – Public Health England[1], the National Institute of Health and Care Excellence[2], American Academy of Pediatrics[3].
We have not based the definition of the routine CESoN on just one set of guidelines because there are subtle differences and gaps in each. We consider the use of all three guidelines necessary to define the 'core' recommendations for the routine CESoN, and this permits a broader swathe of high-quality consensus-based evidence from standard setting organisations (including CPS[4] and HSE[5]) to be considered. It is hoped that a broader definition of the 'core' elements of the CESoN increases the relevance of this scoping review to an international readership of healthcare professionals.

Commented [A3]: Tables columns reordered to reflect reordering of references in the introductory paragraph of the main body.
Information in each column also reordered to help the reader more clearly identify the overlapping recommendations for certain elements of the CESoN from PHE, NICE, and AAP.

Confidential: For Review Only

	Heart: Observation of tone, colour, size and shape of chest, respiratory rate and effort. Palpation of femoral and brachial pulse, capillary refill time, chest, liver. Auscultation of pulmonic, aortic, tricuspid, mitral, and midscapulae/coarctation areas.	heart: position, heart rate, rhythm and sounds, murmurs and femoral pulse volume	
		Lungs: respiratory effort, rate and lung sounds	
		abdomen: assess shape and palpate to identify any organomegaly; check condition of umbilical cord	
	(In males) Testes: Observe the scrotum for symmetry, size and colour. Palpation of scrotal sac (+inguinal canal and perineum if testis not located in scrotal sac).	Genitalia and anus: completeness and patency and undescended testes in boys	No evidence of excessive bleeding at the circumcision site at least 2 hours.
		Spine: inspect and palpate bony structures and check integrity of the skin	
		Skin: colour and texture as well as any birthmarks or rashes	
		Central nervous system: tone, behaviour, movements and posture; check newborn reflexes only if concerned	
	Hips: Observation of leg length, symmetry. Manipulation of each hip with Barlow and Ortolani test manoeuvres.	Hips: symmetry of the limbs, Barlow and Ortolani's manoeuvres	
		Measure weight and head circumference of babies in the first week	weigh the infant
**Relay Examination Findings, Baby Health Advice, Document Procedure and Findings.**		Advice on identifying and managing jaundice	Appropriate jaundice management and/or follow-up plans have been instituted
		Carry out/check bloodspot and hearing screening performed	Newborn metabolic and hearing screenings have been

Confidential: For Review

			completed per hospital protocol
			Newborn pulse oximetry screening has been completed per hospital protocol
		Information to parents on baby care topics including skin, cord, feeding, bonding, signs and symptoms of illness, prevention of SIDS, Vitamin D supplementation, and immunisations.	training and information to mother about: breastfeeding, baby urine and stool patterns, baby care including cord, use of thermometer, signs symptoms of unwell baby, Immunisation, infant safety including car seat usage, prevention of SIDS, and hand hygiene

Confidential: For Review

Appendix II Reporting Guidelines PRISMA ScR Checklist (17)

SECTION	ITEM	PRISMA-ScR CHECKLIST ITEM	REPORTED ON PAGE #
**TITLE**			
Title	1	Identify the report as a scoping review.	1
**ABSTRACT**			
Structured summary	2	Provide a structured summary that includes (as applicable): background, objectives, eligibility criteria, sources of evidence, charting methods, results, and conclusions that relate to the review questions and objectives.	1
**INTRODUCTION**			
Rationale	3	Describe the rationale for the review in the context of what is already known. Explain why the review questions/objectives lend themselves to a scoping review approach.	2
Objectives	4	Provide an explicit statement of the questions and objectives being addressed with reference to their key elements (e.g., population or participants, concepts, and context) or other relevant key elements used to conceptualize the review questions and/or objectives.	2
**METHODS**			
Protocol and registration	5	Indicate whether a review protocol exists; state if and where it can be accessed (e.g., a Web address); and if available, provide registration information, including the registration number.	2
Eligibility criteria	6	Specify characteristics of the sources of evidence used as eligibility criteria (e.g., years considered, language, and publication status), and provide a rationale.	3
Information sources*	7	Describe all information sources in the search (e.g., databases with dates of coverage and contact with authors to identify additional sources), as well as the date the most recent search was executed.	3,4
Search	8	Present the full electronic search strategy for at least 1 database, including any limits used, such that it could be repeated.	4,5
Selection of sources of evidence†	9	State the process for selecting sources of evidence (i.e., screening and eligibility) included in the scoping review.	5
Data charting process‡	10	Describe the methods of charting data from the included sources of evidence (e.g., calibrated forms or forms that have been tested by the team before their use, and whether data charting was done independently or in duplicate) and any processes for obtaining and confirming data from investigators.	5
Data items	11	List and define all variables for which data were sought and any assumptions and simplifications made.	5,6
Critical appraisal of individual sources of evidence§	12	If done, provide a rationale for conducting a critical appraisal of included sources of evidence; describe the methods used and how this information was used in any data synthesis (if appropriate).	6
Synthesis of results	13	Describe the methods of handling and summarizing the data that were charted.	Click here to enter text.
**RESULTS**			

4

Confidential: For Review Only

SECTION	ITEM	PRISMA-ScR CHECKLIST ITEM	REPORTED ON PAGE #
Selection of sources of evidence	14	Give numbers of sources of evidence screened, assessed for eligibility, and included in the review, with reasons for exclusions at each stage, ideally using a flow diagram.	Click here to enter text.
Characteristics of sources of evidence	15	For each source of evidence, present characteristics for which data were charted and provide the citations.	Click here to enter text.
Critical appraisal within sources of evidence	16	If done, present data on critical appraisal of included sources of evidence (see item 12).	Click here to enter text.
Results of individual sources of evidence	17	For each included source of evidence, present the relevant data that were charted that relate to the review questions and objectives.	Click here to enter text.
Synthesis of results	18	Summarize and/or present the charting results as they relate to the review questions and objectives.	Click here to enter text.
**DISCUSSION**			
Summary of evidence	19	Summarize the main results (including an overview of concepts, themes, and types of evidence available), link to the review questions and objectives, and consider the relevance to key groups.	Click here to enter text.
Limitations	20	Discuss the limitations of the scoping review process.	Click here to enter text.
Conclusions	21	Provide a general interpretation of the results with respect to the review questions and objectives, as well as potential implications and/or next steps.	Click here to enter text.
**FUNDING**			
Funding	22	Describe sources of funding for the included sources of evidence, as well as sources of funding for the scoping review. Describe the role of the funders of the scoping review.	7

Confidential: For Review Only

Appendix III: Search strategy for both PubMed and CINAHL

Limits: *Years: 2013-2023; English language; Humans*

PubMed 2023 earch strings keywords (abstract or title) and MeSH SHs:

P: “baby” OR “babies” OR “newborn” OR “newborns” OR “new-born” OR “new-borns” OR “neonate” OR “neonates” OR “neonatal” OR “infant” OR “infants” OR (MM "Infant, Newborn+") OR "infant, newborn"[MeSH Terms] OR "baby"[Title/Abstract] OR "babies"[Title/Abstract] OR "newborn"[Title/Abstract] OR "newborns"[Title/Abstract] OR "new-born"[Title/Abstract] OR "new-borns"[Title/Abstract] OR "neonate"[Title/Abstract] OR "neonates"[Title/Abstract] OR "neonatal"[Title/Abstract] OR "infant"[Title/Abstract] OR "infants"[Title/Abstract]

Concept string 1: "newborn infant physical examination" OR "NIPE" OR “head-to-toe” OR “head to toe” OR “clinical check-up” OR “check-up” OR “newborn exam*” OR “physical examination” OR “discharge examination” OR “examination before discharge” OR “clinical examination” OR “physical assessment” OR “physical check-up” OR "clinical assessment” OR (MM "Physical Examination+") OR ("Physical Examination/instrumentation"[Majr] OR "Physical Examination/methods"[Majr] OR "Physical Examination/standards"[Majr]) OR "newborn infant physical examination"[Title/Abstract] OR "NIPE"[Title/Abstract] OR "head-to-toe"[Title/Abstract] OR "check-up"[Title/Abstract] OR "checkup"[Title/Abstract] OR "newborn examination"[Title/Abstract] OR "physical examination"[Title/Abstract] OR "discharge examination"[Title/Abstract] OR "detailed physical"[Title/Abstract] OR "clinical examination"[Title/Abstract] OR "physical assessment"[Title/Abstract] OR "physical check-up"[Title/Abstract] OR "clinical assessment"[Title/Abstract] OR "developmental dysplasia of the hip/diagnosis"[MeSH Major Topic] OR "cataract/congenital"[MeSH Major Topic] OR "heart defects, congenital/diagnosis"[MeSH Major Topic] OR "cryptorchidism/diagnosis"[MeSH Major Topic] OR "neurologic examination/methods"[MeSH Major Topic] OR "Body Height"[MeSH Major Topic] OR "megalencephaly/diagnosis"[MeSH Major Topic] OR "microcephaly/diagnosis"[MeSH Major Topic] OR "Jaundice, Neonatal/diagnosis"[Majr]

Concept string 2: “performance” OR “procedural” OR “psychomotor skills” OR “technical performance” OR “technical skill” OR “evidence-based” OR “gold-standard” OR “gold standard” OR guidance OR guideline* OR standard* OR checklist OR consensus OR recommendation* OR error* OR omission OR mistake* OR "Psychomotor Performance"[Mesh] OR “Clinical Competence"[Mesh] OR "Evidence-Based Practice"[Mesh] OR "Psychomotor Performance"[MeSH Terms] OR "Clinical Competence"[MeSH Terms] OR "Evidence-Based Practice"[MeSH Terms] OR "Medical Errors"[MeSH Terms] OR "Guideline Adherence"[MeSH Terms] OR "Professional Competence/standards"[Mesh] OR "Standard of Care"[MeSH Terms] OR "performance"[Title/Abstract] OR "procedural"[Title/Abstract] OR "psychomotor skill*"[Title/Abstract] OR "technical performance"[Title/Abstract] OR "technical skill*"[Title/Abstract] OR "technique*"[Title/Abstract] OR "step*"[Title/Abstract] OR "evidence-based"[Title/Abstract] OR "gold-standard*"[Title/Abstract] OR "gold standard*"[Title/Abstract] OR guidance[Title/Abstract] OR guideline*[Title/Abstract] OR standard*[Title/Abstract] OR checklist*[Title/Abstract] OR consensus[Title/Abstract] OR recommendation*[Title/Abstract] OR error*[Title/Abstract] OR omission*[Title/Abstract] OR mistake*[Title/Abstract]

Confidential: For Review Only

Context-

CINAHL 2023 search strings keywords (abstract or title) and MeSH SHs:

Person: TI “baby” OR “babies” OR “newborn” OR “newborns” OR “new-born” OR “new-borns” OR “neonate” OR “neonates” OR “neonatal” OR “infant” OR “infants” OR (MM "Infant, Newborn+") OR AB “baby” OR “babies” OR “newborn” OR “newborns” OR “new-born” OR “new-borns” OR “neonate” OR “neonates” OR “neonatal” OR “infant” OR “infants” OR (MM "Infant, Newborn+")

Concept string 1 : TI "newborn infant physical examination" OR "NIPE" OR “head-to-toe” OR “head to toe” OR “clinical check-up” OR “check-up” OR “newborn exam*” OR “physical examination” OR “discharge examination” OR “examination before discharge” OR “clinical examination” OR “physical assessment” OR “physical check-up” OR "clinical assessment” OR (MM "Neonatal Assessment+") OR (MM "Physical Examination+") OR (MH "Developmental Dysplasia of the Hip/DI") OR (MH "Cataract/DI") OR (MH "Heart Defects, Congenital+/DI") OR (MH "Cryptorchidism/DI") OR (MH "Neurologic Examination/MT") OR (MH "Anthropometry/MT") OR (MH "Head Circumference/MT") OR (MH "Body Height/MT") OR (MH "Microcephaly/DI") OR (MH "Jaundice, Neonatal") OR AB "newborn infant physical examination" OR "NIPE" OR “head-to-toe” OR “head to toe” OR “clinical check-up” OR “check-up” OR “newborn exam*” OR “physical examination” OR “discharge examination” OR “examination before discharge” OR “clinical examination” OR “physical assessment” OR “physical check-up” OR "clinical assessment” OR (MM "Neonatal Assessment+") OR (MM "Physical Examination+") OR (MH "Developmental Dysplasia of the Hip/DI") OR (MH "Cataract/DI") OR (MH "Heart Defects, Congenital+/DI") OR (MH "Cryptorchidism/DI") OR (MH "Neurologic Examination/MT") OR (MH "Anthropometry/MT") OR (MH "Head Circumference/MT") OR (MH "Body Height/MT") OR (MH "Microcephaly/DI") OR (MH "Jaundice, Neonatal")

Concept string 2 : TI “performance” OR “procedural” OR “psychomotor skills” OR “technical performance” OR “technical skill” OR “evidence-based” OR “gold-standard” OR “gold standard” OR guidance OR guideline* OR standard* OR checklist OR consensus OR recommendation* OR error* OR omission OR mistake* OR (MM "Psychomotor Performance+") OR (MM "Professional Competence+") OR (MM "Clinical Competence+") OR (MH "Professional Practice, Evidence-Based+") OR (MM "Health Care Errors+") OR (MM "Practice Guidelines") OR (MM "Guideline Adherence") OR AB “performance” OR “procedural” OR “psychomotor skills” OR “technical performance” OR “technical skill” OR “evidence-based” OR “gold-standard” OR “gold standard” OR guidance OR guideline* OR standard* OR checklist OR consensus OR recommendation* OR error* OR omission OR mistake* OR (MM "Psychomotor Performance+") OR (MM "Professional Competence+") OR (MM "Clinical Competence+") OR (MH "Professional Practice, Evidence-Based+") OR (MM "Health Care Errors+") OR (MM "Practice Guidelines") OR (MM "Guideline Adherence")

Context-

Confidential: For Review Only

:

Confidential: For Review Only

Appendix IV Data Extraction Template - Coding Framework Headings and Tables

CESoN Procedural Elements Coding

1. Prepare to Examine Neonate

- Timing of examination:
- Introduction by examiner;
- Obtain consent from parent.
- Take a History
  - Maternal
  - Fetal
  - Neonatal
- Equipment Preparation

2. Head-to-Toe Physical Examination & Screening of Neonate

- Appearance,
  - including colour, breathing, behaviour, activity and posture
- Head (including fontanelles), face, nose, mouth, ears, neck and general symmetry of head and facial features
  - visual inspection of palate with wooden tongue depressor and torch
  - digital palpation of hard and soft palate
- Eyes
  - Position, colour of sclera
- Eye **screening**:
  - opacities
  - red reflex
- Neck and clavicles,
- Limbs, hands, feet and digits; assess proportions and symmetry
- Heart: rate
- Heart **screening**:
  - position, rhythm and sounds, murmurs and femoral pulse volume, brachial pulse volume
  - Pulse Oximetry: post -ductal
- Lungs: respiratory effort, rate and lung sounds
- Abdomen: assess shape and palpate to identify any organomegaly; check condition of umbilical cord
- Genitalia and anus: completeness and patency
- Genitalia **screening**: undescended testes in boys
- Spine: inspect and palpate bony structures and check integrity of the skin
- Skin: colour and texture as well as any birthmarks or rashes
- Central nervous system:
  - tone, behaviour, movements and posture; check newborn
  - elicit primitive reflexes

Confidential: For Review

- Hips: symmetry of the limbs,
- Hip **screening**:
  - Barlow manoeuvre
  - Ortolani manoeuvre
- Cry: assess sound
- Measure weight and head circumference of babies in the first week and around 8 weeks, and at other times only if there are concerns. Plot the results on the
- Growth chart
- Measure Length

3. Relay Examination Findings, Baby Health Advice, Document Procedure and Findings.

- Parent/Caregiver Concerns
  - Advice on identifying and managing jaundice
  - If there are concerns about the baby's growth
  - Newborn blood spot screening
  - Newborn hearing screening
- Give parents information about:
  - How to bathe their baby and care for their skin
  - Care of the umbilical stump
  - Feeding
  - Bonding attachment
  - How to recognise if the baby is unwell, and how to seek help
  - Established guidance on safer sleeping
  - Maintaining a smoke-free environment for the baby
  - Vitamin D supplements for babies
  - Immunising the baby
- Advise parents to seek advice from a healthcare professional if they think their baby is unwell, and to contact emergency services (call 999) if they think their baby is seriously ill.
  - Consider giving parents information about the Baby Check scoring system
- Referral to specialist

Study Characteristics Coding

- Study Design: RCT, Quasi -experimental, cohort, diagnostic test accuracy, text & opinion
- Publication status: ongoing, completed
- Publication type: journal article, evidence-based guideline, textbook, national handbook/manual, baby health record book
- Study location:
- **Population** -1 (HCP): numbers, discipline/s.
- Population -2 (Neonates): numbers (if provided), gestation, identified risk factors
- **Concept** – CESoN: Lists the head-to-toe elements or focus on specific element
  - Description of procedural element technique
  - Description of incorrect technique
  - Description of optimal timing

Confidential: For Review Only

- **Context** – CESoN
  - setting: hospital, primary care, home
  - timing: early CESoN (within 6 hours of birth), CESoN 6-24 hours following birth, CESoN greater than 24 hours and less than 72 hours following birth, CESoN greater than 72 hours following birth.

Confidential: For Review

Coding Framework Table 1 – Data Extraction Template (colour coding to aid development of evidence map)

Source	Prepare to Examine Neonate	Head-to-Toe Inspection	Screening - Eyes	Screening - Heart	Screening - Hips	Screening - Testes	Primitive Reflexes	Measure HC and Length	Relay Examination Findings, Baby Health Advice, Document Procedure and Findings.
Author/s									
Year									
Geo Location									
Type of Source (Design)<br>1. Experiment<br>2. Cross section/Cohort;<br>3. Observational; 4. Educational;<br>5. Instructional;<br>6. Expert Consensus or Opinion;									
Population –<br>1. Healthy Term Neonate<br>2. At-risk Term neonate									
Population –<br>1. Qualified Practitioner<br>2. Student Learner									
Concept									

**Evidence or Theory focus**<br>**1. full CESoN;**<br>**2. one organ system only OR one screening only),**									
**Context**<br>**1. CESoN timing,**<br>**2. setting;**									

Coding Framework Table 2 – Mapping CESoN to Types of Evidence

**Source**	**Prepare to Examine Neonate**	**Head-to-Toe Inspection**	**Screening - Eyes**	**Screening - Heart**	**Screening - Hips**	**Screening - Testes**	**Primitive Reflexes**	**Measure HC and Length**	**Relay Examination Findings, Baby Health Advice, Document Procedure and Findings.**
Experimental									
Cross section/Cohort;									
Observational;									
Educational;									
Instructional;									
Expert Consensus or Opinion									

Coding Framework Table 3 – Mapping CESoN Recommendations to Jurisdictions

**Source**	**Prepare to Examine Neonate**	**Head-to-Toe Inspection**	**Screening - Eyes**	**Screening - Heart**	**Screening - Hips**	**Screening - Testes**	**Primitive Reflexes**	**Measure HC and Length**	**Relay Examination Findings, Baby Health Advice, Document**

									**Procedure and Findings.**
Ireland									
UK									
USA;									
Canada;									
Australia;									
New Zealand									
Comparison for LMIC: WHO Essentials for P/N Care									

Coding Framework Table 4 – Mapping CESoN Recommendations to descriptions

**Source**	**Prepare to Examine Neonate**	**Head-to-Toe Inspection**	**Screening - Eyes**	**Screening - Heart**	**Screening - Hips**	**Screening - Testes**	**Primitive Reflexes**	**Measure HC and Length**	**Relay Examination Findings, Baby Health Advice, Document Procedure and Findings.**
Description of technique									
Gold-standard technique									
Omissions									
Errors									

PRISMA-P (Preferred Reporting Items for Systematic review and Meta-Analysis Protocols) 2015 checklist: recommended items to address in a systematic review protocol*

Section and topic	Item No	Checklist item	Yes/No/Not applicable (n/a)
**ADMINISTRATIVE INFORMATION**			
Title:			
Identification	1a	Identify the report as a protocol of a systematic review **[scoping]**	Yes
Update	1b	If the protocol is for an update of a previous systematic review, identify as such	n/a
Registration	2	If registered, provide the name of the registry (such as PROSPERO) and registration number	Yes
Authors:			
Contact	3a	Provide name, institutional affiliation, e-mail address of all protocol authors; provide physical mailing address of corresponding author	Yes
Contributions	3b	Describe contributions of protocol authors and identify the guarantor of the review	Yes
Amendments	4	If the protocol represents an amendment of a previously completed or published protocol, identify as such and list changes; otherwise, state plan for documenting important protocol amendments	n/a
Support:			
Sources	5a	Indicate sources of financial or other support for the review	Yes
Sponsor	5b	Provide name for the review funder and/or sponsor	Yes
Role of sponsor or funder	5c	Describe roles of funder(s), sponsor(s), and/or institution(s), if any, in developing the protocol	Yes
**INTRODUCTION**			
Rationale	6	Describe the rationale for the review in the context of what is already known	Yes
Objectives	7	Provide an explicit statement of the question(s) the review will address with reference to participants, interventions, comparators, and outcomes (PICO)	Yes
**METHODS**			
Eligibility criteria	8	Specify the study characteristics (such as PICO, study design, setting, time frame) and report characteristics (such as years considered, language, publication status) to be used as criteria for eligibility for the review	Yes
Information sources	9	Describe all intended information sources (such as electronic databases, contact with study authors, trial registers or other grey literature sources) with planned dates of coverage	Yes
Search strategy	10	Present draft of search strategy to be used for at least one electronic database, including planned limits, such that it could be repeated	Yes

Confidential: For Review Only

Study records:			
Data management	11a	Describe the mechanism(s) that will be used to manage records and data throughout the review	Yes
Selection process	11b	State the process that will be used for selecting studies (such as two independent reviewers) through each phase of the review (that is, screening, eligibility and inclusion in meta-analysis)	Yes
Data collection process	11c	Describe planned method of extracting data from reports (such as piloting forms, done independently, in duplicate), any processes for obtaining and confirming data from investigators	Yes
Data items	12	List and define all variables for which data will be sought (such as PICO items, funding sources), any pre-planned data assumptions and simplifications	Yes
Outcomes and prioritization	13	List and define all outcomes for which data will be sought, including prioritization of main and additional outcomes, with rationale	Yes
Risk of bias in individual studies	14	Describe anticipated methods for assessing risk of bias of individual studies, including whether this will be done at the outcome or study level, or both; state how this information will be used in data synthesis	N/A
Data synthesis	15a	Describe criteria under which study data will be quantitatively synthesised	Y
	15b	If data are appropriate for quantitative synthesis, describe planned summary measures, methods of handling data and methods of combining data from studies, including any planned exploration of consistency (such as I^2, Kendall's τ)	
	15c	Describe any proposed additional analyses (such as sensitivity or subgroup analyses, meta-regression)	
	15d	If quantitative synthesis is not appropriate, describe the type of summary planned	Y
Meta-bias(es)	16	Specify any planned assessment of meta-bias(es) (such as publication bias across studies, selective reporting within studies)	n/a
Confidence in cumulative evidence	17	Describe how the strength of the body of evidence will be assessed (such as GRADE)	Yes

*** It is strongly recommended that this checklist be read in conjunction with the PRISMA-P Explanation and Elaboration (cite when available) for important clarification on the items. Amendments to a review protocol should be tracked and dated. The copyright for PRISMA-P (including checklist) is held by the PRISMA-P Group and is distributed under a Creative Commons Attribution Licence 4.0.**

From: Shamseer L, Moher D, Clarke M, Ghersi D, Liberati A, Petticrew M, Shekelle P, Stewart L, PRISMA-P Group. Preferred reporting items for systematic review and meta-analysis protocols (PRISMA-P) 2015: elaboration and explanation. BMJ. 2015 Jan 2;349(jan02 1):g7647.

Confidential: For Review Only

The procedural elements of the complete examination and screening of the healthy term neonate: a protocol for a scoping review and evidence map

W,W.[1,2], X,X.[1], Y,Y.[1] and Z,Z.[1]

1. University A [blinded for peer review].
2. University B [blinded for peer review].

Confidential: For Review Only

ABSTRACT

Introduction All newborns undergo a **C**omplete **E**xamination and **S**creening **o**f the **N**eonate (CESoN) to verify the general health and wellbeing of the neonate and to screen for signs and symptoms of illness and significant congenital disorders, typically within 72 hours of birth. For healthy, term gestation neonates, this examination is usually performed by a qualified healthcare practitioner i.e., a midwife, nurse, or physician just prior to discharge from the maternity services. As a precursor to modifying and adapting an instrument that measures the quality of performance of the CESoN by healthcare professionals, this review aims to identify, evaluate, synthesise, and map the evidence and theory underpinning current practice and the procedural elements of the CESoN.

Methods and analysis This review will be guided by the Joanna Briggs Institute methodology for scoping reviews, and also the recommendations of the Campbell Collaboration for systematic evidence mapping. Based on the research question, the Person, Concept, Context (PCC) framework will be used to develop eligibility criteria for inclusion in the review. Eligible information shall be sourced by searching electronic databases including PubMed, Cumulative Index of Nursing and Allied Health Literature, and Scopus, and the published guidance from expert bodies on newborn examination and screening (e.g., National Institute and Health and Care Excellence, American Academy of Pediatrics, Royal College of Paediatrics and Child Health), and the grey literature. This study will include primary and secondary research papers, evidence-based guidelines, and expert text and opinion published in English from 2013 to September 2023.

Ethics and Dissemination Ethical approval is not required for this scoping review and systematic evidence mapping. The results from this study will be disseminated through peer-reviewed format i.e., conference proceedings, peer-reviewed healthcare journals.

INTRODUCTION

Rationale

The Complete Examination and Screening of the Neonate (CESoN) is a detailed physical examination of the neonate that is performed within a recommended 72 hours following birth. The CESoN is a separate and more detailed physical examination to that performed within an hour of birth, and typically takes no longer than 15 minutes. The purpose of this detailed physical examination in the healthy term gestation neonate is two-fold. First is to verify the general health and wellbeing of the neonate prior to discharge from the maternity service. The second is to screen the baby for signs and symptoms of specific and significant congenital disorders, which if not detected within the recommended timeframe, may result in severe illness [1-5]. The Newborn and Infant Physical Examination (NIPE) programme [1] describes four of the key screening procedural elements (eyes, heart, hips, testes) of the CESoN. A summary of all the recommended procedural elements of the CESoN, as per the National Institute of Health and Care Excellence [2], Public Health England [1], and the American Academy of Pediatrics [3] is provided (see Appendix I). Optimal performance of the procedural elements of the CESoN is important, particularly the screening elements, given the reality that sub-optimal technique may result in delayed diagnosis and initiation of treatment for progressive congenital disorders [1,6,7]. A 2023 systematic review [8] identified an instrument [9] developed to measure the quality of performance of the complete examination and screening of the neonate by qualified healthcare professionals (HCP).

Objectives

As a precursor to modifying and adapting the identified instrument [9] (which was originally developed and piloted in 2003), this review aims to identify, evaluate, and synthesise the evidence and theory underpinning current practice and procedural elements of this detailed clinical examination. The results of this synthesis and evidence mapping will help guide the future development of standardised practice assessment tools for practitioners of the complete examination and screening of the neonate (CESoN) based on the best available evidence.

The overarching question that this review will attempt to answer is: what are the core and discreet procedural elements of the complete examination and screening of the neonate and their underpinning evidence?

The following sub-questions will structure and focus the data extraction and evidence mapping process:

i. What are the listed discrete elements and core elements outlined within standardised descriptions of the CESoN of the healthy term gestation neonate?

ii. What are the recommended techniques and procedural processes for conducting the various elements of the CESoN of the healthy term gestation neonate?

iii. What are the differences, similarities, variations in suggested practice between standardised descriptions of the CESoN of the healthy term gestation neonate?

iv. What types and quality of evidence underpin recommended best practice of the standardised descriptions of CESoN of the healthy term gestation neonate?

v. What are the omissions, and procedural errors in the conduct of the CESoN of the healthy term gestation neonate?

Confidential: For Review Only

This scoping review is registered with the Open Science Framework: https: XXXXXX [blinded for peer review process]

METHODS AND ANALYSIS:

Leading experts in both scoping review and mapping review methodologies have clarified key differences between these two methodologies [10,11] though it is acknowledged that both types of review have many similarities and overlapping objectives [12].

For the creation of a systematic evidence map, we are referring to the Campbell Collaboration recommendations [13], which cautions researchers that evidence maps "...*summarize what evidence there is, not what the evidence says*".

The review objectives also require us to determine the recommended techniques for conduct of the various procedural elements of the CESoN. Omissions and errors specific to the conduct of the CESoN will also be identified. This goes beyond the above stated evidence mapping review objective of summarising the types of evidence. Therefore, this review must also scope the literature to permit an in-depth analysis of the evidence related to practices and procedural elements specific to the CESoN.

This format of this protocol follows both the author guidelines of the target publication for this protocol [14] and is informed by the recommendations [15] on scoping review protocol items to be reported, and the Preferred Reporting Items for Systematic Review and Meta-Analysis Protocols Checklist (PRISMA-P)[16].

The completed review will be reported using the PRISMA Extension for Scoping Reviews checklist [17] (see Appendix II).

Eligibility Criteria

This review is scoping in nature and seeks to map the evidence underpinning the procedural elements of a multifaceted physical evaluation and screening test performed on all babies within a specific timeframe following birth. The procedural elements of the CESoN are not the type of diagnostic tests (observation, inspection, palpation of the baby's body parts and organ systems) that can be evaluated in effectiveness studies. Therefore, the Population, Intervention, Comparator, Outcome, Study Type (PICOS) mnemonic is not suitable to frame the research question and determine eligibility criteria. Following the recommendations of seminal methodology papers [10,11,15,18] including the scoping review chapter of the JBI Manual for Evidence Synthesis [19], the PCC mnemonic - Participants (P), Concept, (C) and Context (C) is used to define the inclusion criteria for evidence sources. Eligibility criteria must be congruent with the focus and scope of the review question.

Participants (P)

Inclusion criteria for neonates being examined will be a gestation of 37+0 weeks or greater at birth; being born with APGAR score greater than seven at five minutes following birth; being primarily cared for by their parent from birth until their CESoN.

Inclusion criteria for practitioners performing the complete examination and screening of the healthy term gestation neonate include paediatricians, neonatologists, midwives, nurses, general practitioners

Confidential: For Review Only

and clinicians or academics or researchers or experts from those professional disciplines that routinely conduct detailed physical examinations of healthy term gestation neonates.

Exclusion criteria for neonates will include neonates born at less than 37 weeks gestation; neonates admitted to a neonatal unit for ongoing care; neonates born with significant congenital disorders or illness diagnosed antenatally or at birth.

Exclusion criteria for practitioners will include pathologists, ultrasonographers, and other specialists within foetal medicine, as diagnostic imaging or other investigations used for examination and screening of either the foetus or neonate following stillbirth or neonatal death are not relevant.

Concept (C)

The concept is the recommended best practices of the procedural elements of the complete examination and screening of the healthy term gestation neonate.

Inclusion criteria for concept: complete descriptions of the CESoN or the Newborn Infant Physical Examination (NIPE) will be sought. In addition, description of key elements or groups of steps, for example cardiac screening, developmental dysplasia of the hips (DDH) will be sought, provided they are conducted within the context of the CESoN or the Newborn Infant Physical Examination (NIPE).

Evidence underpinning the complete descriptions of the CESoN, or the Newborn Infant Physical Examination (NIPE) will be sought. In addition, evidence underpinning the description of key elements or groups of steps for example cardiac screening, DDH screening will be sought, provided such evidence is collected within the overall context of the CESoN or the Newborn Infant Physical Examination (NIPE).

Context (C)

The context includes the timing and location of the examination. The CESoN may be conducted by a qualified healthcare practitioner in a hospital, primary care, or home setting. The CESoN is a distinct and separate physical examination to the less detailed physical examination of the baby completed within an hour of birth which is typically performed by a midwife [5,20]. Evidence will not be drawn exclusively from high income or English-speaking countries as various procedural elements of the CESoN are conducted by healthcare practitioners from every geographical region [20].

Search Strategy

An initial scoping search using Google Scholar, Google searching, and OneSearch [21] via the University's library search engine was used to iteratively identify relevant keywords and surrogate terms for the core concepts of the search strings using the PCC framework.

The search strategy for the proposed scoping review and evidence mapping will aim to locate both published and unpublished documents using a three-step search strategy. This will involve an initial limited search of both PubMed and CINAHL to identify studies relevant to the topic. Following this initial search, analysis of the text words contained in the titles and abstracts of relevant articles, and the index terms used to describe the articles further informed the development a full search strategy for PubMed (see Appendix III).

Confidential: For Review Only

A second search will then be conducted across all included databases, incorporating all identified keywords and index terms. The databases to be searched include PubMed, CINAHL (EBSCO), Web of Science Core Collection, Scopus, Maternity and Infant Care (MIDIRS), Cochrane Database of Systematic Reviews, Cochrane Central Register of Controlled trials (CENTRAL), and Joanna Briggs Institute Library.

Thirdly, the reference lists of all identified studies and reports will be screened for additional sources. Sources of unpublished and grey literature, Open Grey, and Google Scholar will also be searched using an abridged version of the original search strings. Similarly, particular websites of relevant expert academic and professional societies will be searched to locate updated national guidance on specific aspects of newborn screening e.g., American Academy of Ophthalmology, American Academy of Orthopaedic Surgeons, American Urological Association, Association for European Paediatric and Congenital Cardiology, Royal College of Paediatrics and Child Health. The key grey literature search terms include newborn and physical examination. In websites or sources where there is a large return of citations, the author will use content relevancy tools, where available, and will screen the first 100 citations for potential inclusion.

A time-limit of 10 years will be applied to assessing eligibility for inclusion. Only evidence published in the English-language will be included.

Types of Evidence Sources

Original research, evidence syntheses, clinical practice guidelines, and guidance published by standard-setting organisations or expert professional bodies. In addition, guidance published within comparable healthcare systems in English-speaking countries, U.K., Ireland, Australia, NZ, Canada, U.S.A., and guidance published in the E.U. in the English language will be included.

Select core textbooks published or re-issued from January 2018 with complete descriptions of the procedural elements of the CESoN will be included.

In addition, literature relating to the effectiveness of steps (published within the context of the CESoN) within the procedure at diagnosing anomalies; or detailing errors or omissions in performance of the procedure will also be included.

Data Charting and Synthesis

Coding and Data Extraction

A key characteristic of evidence mapping is predefining coding categories ahead of the data extraction process [22]. The pre-defined coding categories reflect the information required to address the main objectives of the review question. The characteristics of the studies/papers (e.g., source (author, year, country), primary study design and publication type, participant details, evidence underpinning development of description of the CESoN concept studied, context of CESoN, and key findings relevant to the review questions will be extracted (see Appendix IV).

The data extraction form will be piloted on a sample of three studies by two review authors to assess the feasibility and robustness of the data extraction process. The outcome of the pilot phase will determine whether the data extraction form requires further modifications to comprehensively address the research questions. Any deviations from the above will be outlined in the full scoping review report. Data extraction will be performed by one reviewer (WW)and then checked by a second

Confidential: For Review Only

review author (XX or YY). Other review authors will check random samples of data (20% of total papers included) to ensure high-quality data extraction has been achieved. Any disagreements will be resolved by the involvement of a third review author (ZZ).

The pre-defined coding categories are the 'framework' to classify the data for the evidence synthesis. As our review question and objectives do not align with the frameworks applied to standard evidence mapping reviews e.g., 3ie gap maps, Sightsaver 3 EGMs, we will develop a framework based on the procedural elements of the CESoN. These elements are an amalgamation drawn from three institutions – the American Academy of Pediatrics, the National Institute of Health and Care Excellence, and Public Health England. These internationally recognised expert institutions have published recommendations on the CESoN [1,2,3]. Initially the procedural elements and associated omissions and errors extracted from the included papers are mapped to the three broad themes:

1. Preparation of the neonate for the examination
2. Details for the systematic process and procedural steps for the physical examination and screening
3. Documentation, sharing details of the findings of the examination, providing advice and information

Comparisons are drawn across included papers in terms of numbers of steps, sequencing of steps and errors identified. Once all data is extracted, descriptions of each step are compared and condensed. The aim for each step of the process is to iteratively create a summative, salient, and essence-capturing step with a clear beginning and end to a step outlined, based upon the comparison of descriptions provided. Errors associated with each step are also summarised.

In order to address the certain research sub-questions (i, iv), the data extraction coding framework will also include a mapping of the evidence underpinning recommended best practice for discrete steps or groups of steps relating to discrete assessments conducted as part of the overall CESoN. The evidence underpinning a step, or a group of steps is extracted and summarised. Heat maps will be created as cross tabulations of categorical variables, showing:

1. Number of papers itemising each procedural element (i.e., key step or groups of steps)
2. Countries/regions itemising each procedural element (i.e., key step or groups of steps)
3. Volume or strength of evidence for each procedural element (key step or group of steps) of the CESoN.

A narrative summary will accompany the tabulated and/or charted results and will describe how the results relate to the reviews objective and question/s.

Quality Assessment

For the traditional scoping review, an assessment of the strength of the body of evidence using the Grading of Recommendations, Assessment, Development and Evaluation (GRADE) tool is not ordinarily conducted [15].

Seminal papers on scoping review and mapping review methodologies state that critical appraisal of included studies is optional [10,11]. However, other notable papers on mapping review methodology

Confidential: For Review Only

[13,22] recommend critical appraisal of selected included studies to increase confidence in the findings of the evidence synthesis e.g., systematic reviews, primary studies.

For papers which describe the complete CESoN or NIPE the review authors will describe the processes which underpinned the development of the description or guidance outlining the procedural elements and steps of the CESoN.

The quality of evidence underpinning the elements of CESoN will be assessed, where possible, using quality assessment tools appropriate to each evidence source i.e., primary research, secondary research sources. For example, the quality of any systematic reviews of certain elements of the CESoN (e.g., physical examination and screening of the hips for DDH) will be assessed using the JBI critical appraisal tool for systematic reviews [23] . Where a clinical practice guideline is identified, it will be assessed for methodological quality using appraisal tools such as the CASP checklists and AGREE II checklist [24]. For expert text and opinion sources, the JBI critical appraisal tool will be used [25].

ETHICS AND DISSEMINATION

Ethical approval is not required for this scoping review and systematic evidence mapping. The results from this study will be disseminated through peer-reviewed processes for example conference proceedings, peer-reviewed healthcare journals. The final review will also be included with the doctoral thesis of the lead author (WW) and made publicly available on the university's Open Access institutional repository.

Authors' contributions:

WW, XX, YY, and ZZ involved in conception & design of review, acquisition & analysis of data, interpretation of findings, drafting and reviewing manuscripts.

Funding statement: 'This research received no specific grant from any funding agency in the public, commercial or not-for-profit sectors'. The doctoral studies of the lead author (WW), of which this work is in part-fulfilment, are funded by their employer (University B).

Competing interests statement The authors of this paper have no competing interests to declare.

What is already known on this topic

The Complete Examination and Screening of the Neonate (CESoN) is an important, universal baseline assessment of newborn health and wellbeing. Despite its importance, there is a paucity of research on many of the psychomotor and assessment skills utilised by clinicians to conduct the CESoN.

What this study hopes to add

This study hopes to provide clarity on the both the sources and levels of evidence underpinning recommendations for optimal performance of the CESoN. This scoping review is part of an evidence mapping process to inform modifications that may be needed to update and pilot an instrument that measures clinician performance of the CESoN.

How this study might affect research, practice or policy

Confidential: For Review Only

This study may positively affect practice by providing clinicians and academics with an evidence map that illustrates the strength of evidence underpinning the identified performance elements of the CESoN. As the ultimate goal of the CESoN is to protect neonatal health by detecting early signs and symptoms that may indicate illness or congenital disorders, it is vital that the examination and screening techniques are aligned to the latest and best level-evidence available.

Confidential: For Review Only

Keywords

Newborn; Physical Examination; Psychomotor Performance; Evidence-Based Practice.

Acknowledgements

I wish to thank AA [blinded for peer review] for his invaluable support and contributions since joining my doctoral supervision panel in December 2023. A very special acknowledgement and sincere gratitude to my supervisor and co-author XX upon her recent retirement from [University A]. This review is to contribute towards a doctoral degree award for author WW [blinded for peer review process].

Confidential: For Review Only

References

1. Public Health England (PHE) Newborn and infant physical examination (NIPE) screening programme handbook. *1. NIPE screening examination*. PHE. 2021. https://www.gov.uk/government/publications/newborn-and-infant-physical-examination-programme-handbook/newborn-and-infant-physical-examination-screening-programme-handbook#training-and-competency (accessed 01 July 2023).
2. National Institute of Health and Care Excellence (NICE) Postnatal Care NICE guideline NG194. *1.3 Postnatal care of the baby*. NICE. 2021. 18-21. https://www.nice.org.uk/guidance/ng194 (accessed 01 July 2023).
3. Benitz, W.E. et al. 2015. Hospital stay for healthy term newborn infants. *Pediatrics (Evanston)* 135(5) 948-953. DOI: 10.1542/peds.2015-0699
4. Lemyre B, Jefferies AL, O'Flaherty P. Facilitating discharge from hospital of the healthy term infant. Paediatrics & child health. 2018;23(8):515-22. DOI: 10.1093/pch/pxy127
5. Health Service Executive (HSE). The Newborn Clinical Examination Handbook [online]. HSE. 2018. https://www.hse.ie/eng/about/who/healthwellbeing/our-priority-programmes/child-health-and-wellbeing/neonate%20exam.pdf (accessed 01 July 2023).
6. Paton RW. Screening in Developmental Dysplasia of the Hip (DDH). The surgeon (Edinburgh). 2017;15(5):290-6. DOI: 10.1016/j.surge.2017.05.002
7. Martin GR, Ewer AK, Gaviglio A, Hom LA, Saarinen A, Sontag M, et al. Updated Strategies for Pulse Oximetry Screening for Critical Congenital Heart Disease. Pediatrics. DOI: 10.1542/peds.2019-16502020;146(1):e20191650.
8. BLINDED for peer review process
9. Townsend, J. et al. 2004. Routine examination of the newborn: the EMREN study. Evaluation of an extension of the midwife role including a randomised controlled trial of appropriately trained midwives and paediatric senior house officers - Introduction. *Health technology assessment (Winchester, England)* 8(14) 1. DOI: 10.3310/hta8140
10. Campbell F, Tricco AC, Munn Z, Pollock D, Saran A, Sutton A, et al. Mapping reviews, scoping reviews, and evidence and gap maps (EGMs): the same but different- the "Big Picture" review family. Syst Rev. 2023;12(1):45. DOI: 10.1186/s13643-023-02178-5
11. Khalil H, Tricco AC. Differentiating between mapping reviews and scoping reviews in the evidence synthesis ecosystem. Journal of clinical epidemiology. 2022;149:175-82. DOI: 10.1016/j.jclinepi.2022.05.012
12. Chen Y-F, Takwoingi Y, Grove A. Mapping review could be seen as a subtype of scoping review and differentiating between the action of mapping evidence and presentation of evidence as maps may be helpful: response to Khalil et al. Journal of clinical epidemiology. 2023;157:110-1. DOI: 10.1016/j.jclinepi.2023.02.017
13. Saran A, White H. Evidence and gap maps: a comparison of different approaches. Campbell Systematic Reviews. 2018;14(1):1-38. https://doi.org/10.4073/cmdp.2018.2
14. BMJ Paediatrics Open. Submission Guidelines online: BMJ Publishing & RCPCH; 2023. https://bmjpaedsopen.bmj.com/pages/authors/ (accessed 15 August 2023).
15. Peters MDJ, Godfrey C, McInerney P, et alBest practice guidance and reporting items for the development of scoping review protocols. JBI Evidence Synthesis. 2022;20(4):953-68. DOI: 10.11124/jbies-21-00242
16. Moher D, Shamseer L, Clarke M, et alPreferred reporting items for systematic review and meta-analysis protocols (PRISMA-P) 2015 statement. Systematic Reviews. 2015;4(1):1. DOI: 10.1186/2046-4053-4-1

Confidential: For Review Only

17. Tricco A.C. LE, Zarin W, O'Brien K.K, et alPRISMA Extension for Scoping Reviews (PRISMA-ScR): Checklist and Explanation. Annals of Internal Medicine. 2018;169(7):467-73. https://doi.org/10.7326/M18-0850
18. Khalil H, Peters MDJ, Tricco AC, Pollock D, Alexander L, McInerney P, et al. Conducting high quality scoping reviews-challenges and solutions. Journal of Clinical Epidemiology. 2021;130:156-60. https://doi.org/10.1016/j.jclinepi.2020.10.009
19. Aromataris E, Munn Z (Editors). *JBI Manual for Evidence Synthesis.* JBI, 2020. Available from https://synthesismanual.jbi.global. https://doi.org/10.46658/JBIMES-20-01
20. W.H.O. Pregnancy, childbirth, postpartum and newborn care: A guide for essential practice (3rd edition) (PCPNC). 2015. Newborn Care. Geneva, World Health Organisation. 184. https://www.ncbi.nlm.nih.gov/books/NBK326678/ (accessed 01 July 2023).
21. UCC Library (2023) What is OneSearch and how do I use it? https://askus.booleweb.ucc.ie/research/faq/190571#:~:text=OneSearch%20is%20a%20single%2Dsearch,on%20the%20UCC%20Library%20homepage (accessed 15 August 2023).
22. White, H. et al. 2020. Guidance for producing a Campbell evidence and gap map. *Campbell Syst Rev* 16(4) e1125. DOI: 10.1002/cl2.1125
23. Aromataris, E. et al. 2015. Summarizing systematic reviews: methodological development, conduct and reporting of an umbrella review approach. *JBI Evidence Implementation* 13(3) 132-140. DOI: 10.1097/xeb.0000000000000055
24. Brouwers, M.C. et al. 2010. AGREE II: advancing guideline development, reporting and evaluation in health care. *Canadian Medical Association journal (CMAJ)* 182(18) E839-842. DOI: 10.1503/cmaj.090449
25. McArthur A, K.J., Yan H, Florescu S. 2020. JBI Critical Appraisal Checklist for Text and Opinion Papers. *In* M. Z. Aromataris E ed. *JBI Manual for Evidence Synthesis*. Joanna Briggs Institute. https://doi.org/10.46658/JBIMES-20-05

Confidential: For Review Only
